# A high-volume study on the impact of diabetes mellitus on clinical outcomes after surgical and percutaneous cardiac interventions

**DOI:** 10.1186/s12933-024-02356-2

**Published:** 2024-07-18

**Authors:** S. R. Slingerland, D. N. Schulz, G. J. van Steenbergen, M. A. Soliman-Hamad, J. M. H. Kisters, M. Timmermans, K. Teeuwen, L. Dekker, D. van Veghel, On behalf of the participating centres of the Cardiothoracic surgery registration committee, the PCI registration committee and the THI registration committee of the Netherlands Heart Registration (NHR)

**Affiliations:** 1 Catharina Heart Centre, Catharina hospital, P.O. Box 1350, 5602 ZA Eindhoven, The Netherlands; 2 Department of Biomedical Technology, Eindhoven University of Technology, 5612 AZ Eindhoven, The Netherlands; 3 Netherlands Heart Registration, Moreelsepark 1, 3511 EP Utrecht, The Netherlands; 4 Department of Cardiology, Catharina hospital, P.O. box 1350, 5602 ZA Eindhoven, The Netherlands

**Keywords:** Cardiovascular disease, Endocrinology, Percutaneous coronary intervention, Coronary artery bypass grafting, Aortic valve replacement, Transcatheter aortic valve implantation

## Abstract

**Background:**

Type I and type II diabetes mellitus (DM) patients have a higher prevalence of cardiovascular diseases, as well as a higher mortality risk of cardiovascular diseases and interventions. This study provides an update on the impact of DM on clinical outcomes, including mortality, complications and reinterventions, using data on percutaneous and surgical cardiac interventions in the Netherlands.

**Methods:**

This is a retrospective, nearby nationwide study using real-world observational data registered by the Netherlands Heart Registration (NHR) between 2015 and 2020. Patients treated for combined or isolated coronary artery disease (CAD) and aortic valve disease (AVD) were studied. Bivariate analyses and multivariate logistic regression models were used to evaluate the association between DM and clinical outcomes both unadjusted and adjusted for baseline characteristics.

**Results:**

241,360 patients underwent the following interventions; percutaneous coronary intervention(*N* = 177,556), coronary artery bypass grafting(*N* = 39,069), transcatheter aortic valve implantation(*N* = 11,819), aortic valve replacement(*N* = 8,028) and combined CABG and AVR(*N* = 4,888). The incidence of DM type I and II was 21.1%, 26.7%, 17.8%, 27.6% and 27% respectively. For all procedures, there are statistically significant differences between patients living with and without diabetes, adjusted for baseline characteristics, at the expense of patients with diabetes for 30-days mortality after PCI (OR = 1.68; *p* <.001); 120-days mortality after CABG (OR = 1.35; *p* <.001), AVR (OR = 1.5; *p* <.03) and CABG + AVR (OR = 1.42; *p* =.02); and 1-year mortality after CABG (OR = 1.43; *p* <.001), TAVI (OR = 1.21; *p* =.01) and PCI (OR = 1.68; *p* <.001).

**Conclusion:**

Patients with DM remain to have unfavourable outcomes compared to nondiabetic patients which calls for a critical reappraisal of existing care pathways aimed at diabetic patients within the cardiovascular field.

**Supplementary Information:**

The online version contains supplementary material available at 10.1186/s12933-024-02356-2.

## Introduction

Over the years, the prevalence of diabetes mellitus (DM) is increasing as a result of ageing and lifestyle habits [1–6]. Both type I and II DM are associated with amongst others cardiovascular diseases (CVD) [1, 2, 7, 8]. Due to these co-morbidities, people with diabetes consume more care which is associated with higher healthcare costs compared to people without diabetes [3, 9, 10].

Cardiovascular disease is a common cause of death for people with diabetes [2, 9, 11, 12]. Earlier research showed that people with diabetes undergoing surgical and percutaneous cardiac procedures, compared to people without diabetes, are at higher risk of mortality, reinterventions, and complications like deep sternal wound infections (DSWI), stroke and kidney failure [3, 8, 13–15]. As the disease burden and healthcare consumption will further rise in the coming years due to the doubling of diabetes prevalence, care for patients with diabetes constitutes an ongoing concern [9, 11, 16]. Therefore, healthcare protocols, clinical pathways and drug treatment options in both primary and secondary (cardiac) care are continuously adjusted for patients with diabetes to optimize health outcomes [16, 17].

However, we lack recent, real-world data to evaluate outcomes in patients with diabetes. This study aims to assess the impact of diabetes mellitus on present-day clinical outcomes in patients undergoing surgical and percutaneous cardiac procedures in the Netherlands.

## Method

### Study design

This is a retrospective study using observational, real-world data from all hospitals participating in the voluntary public reporting program facilitated by the Netherlands Heart Registration (NHR) [18, 19]. This includes 14 out of 16 Dutch heart centres regarding CABG, TAVI, AVR and CABG + AVR, 21 out of 30 centres that perform PCI procedures (of which 14 do not have cardiothoracic surgery onsite) and 30 out of 30 centres that perform PCI after 2017. The NHR is a national mandatory quality registration in which hospitals register a standard set of baseline, procedural and outcome data of all invasive cardiac procedures. The NHR annually publishes outcome data of participating hospitals to improve the quality, safety and transparency of care in a voluntary reporting program [18, 19]. In the voluntary public reporting program, each hospital is responsible for the active follow-up per patient and each hospital must actively check for reinterventions and outcomes after discharge. The registered data and the data collection process are checked by the NHR, amongst others in annual audits [20].

### Inclusion criteria

The study population consisted of all patients with CVD, treated with an invasive cardiac intervention for coronary artery disease (CAD), aortic valve disease (AVD), or combined CAD and AVD between January 1st 2015 and December 31st 2020. Patients undergoing the following cardiac procedures are included: percutaneous coronary intervention (PCI), coronary artery bypass grafting (CABG), aortic valve replacement (AVR), transcatheter aortic valve implantation (TAVI) and combined CABG and AVR.

Per invasive cardiac intervention, only the first CABG, AVR, TAVI or CABG + AVR per patient in the period 2015–2020 is included. Likewise, if a patient underwent multiple PCIs within one year, only the first PCI (within 365 days) is included in the dataset. A second procedure within one year was excluded or was considered a reintervention if the procedure meets the NHR-definition of reintervention.

### Baseline characteristics and outcome measures

The baseline characteristics and outcome measures (mortality, complications and reinterventions) per invasive cardiac intervention are presented in Table 1 in the supplementary materials. These variables are standard parameters within the NHR [18, 19, 21].

### Definitions

Diabetes mellitus (DM) is defined as having chronic hyperglycaemia, diagnosed prior to cardiac intervention, demonstrating one of the following criteria; (1) fasting plasma glucose ≥ 7.0 mmol/l (2) 2-hour post-load (75 mg glucose) plasma glucose ≥ 11.1 mmol/l (3) symptoms of hyperglycaemia and plasma glucose ≥ 11.1 mmol/l and (4) glycated haemoglobin (HbA1c) ≥ 6.5% [21–23]. If a patient is considered diabetic, the treatment method is registered as well: insulin, oral medication, diet, other treatment, no treatment or treatment unknown.

The following six other variables are worth defining; an urgent procedure, myocardial infarction (MI), cerebrovascular accident (CVA) with a residual deficit, re-exploration, DSWI, and Target Vessel Revascularization (TVR).

First, urgent procedures are patients who are not electively admitted for surgery but for medical reasons require intervention within the current admission [21].

Second, MI is defined as an increase and subsequent decrease in one or more biomarkers (preferably troponin) by at least one value above the 99th percentile of the upper limit where at least one of the following symptoms is present; (1) symptoms appropriate to ischemia (2) new significant ST-segment or T-wave abnormalities or bundle branch block (3) development of pathological waves on the electrocardiogram (4) new loss of viable myocardial tissue or new wall motion abnormalities demonstrated by imaging technologies and/or (5) identification of intracoronary thrombus on angiography or autopsy [21, 24, 25].

Third, CVA with residual deficit during hospital admission is a permanent, neurological dysfunction as determined by a neurologist due to focal ischemia of the brain, spinal cord or retina caused by acute infarction of the neurological tissue due to thrombosis, embolism, systemic hypoperfusion or haemorrhage [21, 26].

Fourth, re-exploration within 30 days after the intervention is defined as the first rethoracotomy after the initial closing of the thorax due to bleeding, tamponade or cardiac problems [21].

Fifth, DSWI within 30 days after the intervention covers muscle, sternum and mediastinum and is positive if one or more of the following criteria applies: (1) surgical drainage or fixation sternum due to deep sternal wound (2) positive wound cultures and (3) antibiotics-therapy for sternum wound treatment [21].

Last, TVR within one year after the intervention is defined as revascularization by PCI in the same vessel (or vessels) that had been treated at the index PCI within 1 year (≤ 365 days) of the index PCI, or; revascularization by CABG in the same vessel (or vessels) that had been treated at the current PCI after 1 day and within 1 year (≤ 365 days) of the PCI. An urgent CABG performed within 1 day of the current PCI is recorded as an outcome indicator of urgent CABG and not as an occurrence of TVR [21].

The definitions of other included variables are aligned with the European Society of Cardiology’s (ESC) Guidelines [18].

### Statistical analyses

Following current guidelines on the imputation of data by The Dutch Journal of Medicine (2013) and BMC Medical Research Methodology (2017), missing values of baseline characteristics were imputed [27, 28]. The baseline characteristics, that are missing at random and missing less than 35%, were imputed using ten iterations and ten imputations using both baseline characteristics and outcomes as predictors [27, 28]. An overview of all available variables and the rate of completeness can be found in Table 1 of the Supplementary materials. Outcome variables only served as predictors and were not imputed. Using descriptive statistics, QQ-plots and Kolmogorov-Smirnov tests, the structure of the dataset was studied and checked for outliers and normal distribution. Continuous variables are shown as mean (standard deviation (SD)) or median (interquartile range (IQR)) while categorical variables are shown as absolute and relative frequencies. Bivariate analyses were performed to study differences between patients with diabetes and patients without diabetes for baseline characteristics and outcome measures using Mann-Whitney U and Pearson χ2- tests. Logistic regression analyses were used to analyse the association between diabetes mellitus and short-term outcome measures while adjusting for all applicable baseline characteristics and the year of intervention. An overview of the applicable baseline characteristics per intervention that were used in the logistic regression analyses is presented in Table 1 in the Supplementary materials. Per invasive cardiac intervention, for each (short-term) outcome measure, the odds ratio (OR) and p-value for diabetes are shown. Furthermore, two additional logistic regressions were performed as sensitivity analyses; one analysis while making cohorts for the year of intervention (2015–2017 versus 2018–2020) to study differences in time and one by making cohorts for the different treatment of diabetes (as seen in Supplementary Table 2 and as defined above) for each intervention [21–23]. Furthermore, a propensity score (PS) matching analysis was executed to study if we adequately adjusted for differences in baseline characteristics. The PS included all baseline characteristics listed in Table 1. For each patient with DM, a propensity score matched patient without DM was selected (ratio 1:n) using the nearest neighbour (with a calliper width of 0.2 of the pooled SD of the logit of the propensity score) and no replacement. Covariate balance was evaluated with standardised mean differences, and a standardised mean difference < 0.1 was considered a negligible difference between cohorts.

Last, to study the relationship between patients with diabetes and patients without diabetes in relation to survival and reintervention during follow-up, Cox regression curves with 95% CI were used while correcting for baseline characteristics (as seen in Table 1 in Supplementary materials). The proportional hazards assumption was checked by plotting a log minus log survival curve stratified for each covariate per intervention.

Results for all analyses were considered statistically significant with a two-tailed p-value < α 0.05. All analyses were performed using SPSS 29 (SPP Inc., Chicago IL, USA) and Rstudio (Rstudio Inc., Boston, MA, USA).

## Results

A total of 241,360 invasive cardiac interventions were analysed, divided among the following interventions: PCI (*N* = 177,556), CABG (*N* = 39,069), AVR (*N* = 8,028), TAVI (*N* = 11,819), and CABG + AVR (*N* = 4,888). Of all patients undergoing PCI, 21.1% has diabetes, 26.7% of CABG patients, 17.8% of AVR, 27.6% of TAVI and 27.8% of CABG + AVR. Oral medication is the most common medical treatment for DM, ranging between 6.7 − 14.4% of patients with diabetes, and subsequently insulin treatment, ranging between 4.2 − 9.7% of patients with diabetes. More information regarding diabetes within the study population is presented in Table 2 in the Supplementary Materials.

Table 1 shows descriptive and univariable analyses of baseline characteristics between patients with diabetes and patients without diabetes for each cardiac procedure. All baseline characteristics within the PCI cohort are statistically significantly different between patients with diabetes and patients without diabetes (*p* <.001). For CABG there is a statistically significant difference for all baseline characteristics (*p* <.001) except for urgency of procedure (*p* =.112). In the AVR cohort, the variables age, eGFR, body mass index (BMI) and chronic obstructive pulmonary disease (COPD), logistic EuroSCORE I, logistic EuroSCORE II (*p* <.001) and CVA (*p* =.03) were significantly different between the two groups. Regarding TAVI, there is a significant difference for all baseline characteristics (*p* <.001) except EuroSCORE I (*p* =.112). Lastly, the CABG + AVR cohort shows a statistically significant difference in age, eGFR, BMI, chronic lung disease, EuroSCORE II (*p* <.001) and left ventricular ejection fraction (LVEF) (*p* =.005), multivessel disease (*p* =.016), prior myocardial infarction (*p* =.043) and EuroSCORE I (*p* =.004).

**Table 1:** Univariable analyses of patient characteristics between patients living with diabetes and people living without diabetes for each cardiac procedure

Variable	Coronary artery disease (CAD)						Aortic valve disease (AVD)						Combined CAD and AVD		
	PCI *n* = 177,556			CABG *N* = 39,069			AVR *N* = 8,028			TAVI *N* = 11,819			CABG + AVR *N* = 4,888		
	DM	No DM	*p-*value	DM	No DM	*p-*value	DM	No DM	*p-*value	DM	No DM	*p-*value	DM	No DM	*p-*value
***Sex (male)***,*** n (%)***	*26*,*193 (68.5)*	*101*,*530 (72.9)*	*< 0.001*	*8051 (77.1)*	*23*,*587 (82.5)*	*< 0.001*	*884 (61.9)*	*3*,*954 (60.3)*	*0.327*	*1*,*731 (52.9)*	*4*,*286 (50.2)*	*0.009*	*1*,*035 (76.0)*	*2*,*685 (76.1)*	*0.884*
***Age***,*** median (IQR)***	*70.0 (62.0–76.0)*	*66.0 (57.0–75.0)*	*< 0.001*	*68.0 (61–74)*	*67.0 (60–74)*	*< 0.001*	*71.0 (57.7–85.1)*	*73.1 (61.9–85.0)*	*< 0.001*	*79.0 (74.0–83.0)*	*81.0 (77.0–85.0)*	*< 0.001*	*72.0 (68.0–76.0)*	*73.0 (68.0–78.0)*	*< 0.001*
***eGFR***,*** median (IQR)***	*67.6 (48.1–85.3)*	*73.2 (58-87.3)*	*< 0.001*	*72.3 (57-87.3)*	*74.6 (63.7–86.1)*	*< 0.001*	*70.9 (57.6–85.1)*	*73.1 (61.9–85.1)*	*< 0.001*	*48.4 (35.6–64.2)*	*52.3 (40.0-66.2)*	*< 0.001*	*67.5 (52.7–83.3)*	*71.2 (59.0-82.3)*	*< 0.001*
***LVEF***,*** median (IQR)***	*55.0 (40.0–55.0)*	*55.0 (40.0–55.0)*	*< 0.001*	*50.0 (40.0–55.0)*	*55.0 (50.0–55.0)*	*< 0.001*	*55.0 (55.0–60.0)*	*55.0 (55.0–60.0)*	*0.741*	*55.0 (40.0–55.0)*	*55.0 (43.0–55.0)*	*< 0.001*	*55.0 (43.0–56.0)*	*55.0 (50.0–56.0)*	*0.005*
***BMI***,*** median (IQR)***	*n/a*	*n/a*	*n/a*	*28.4 (25.7–31.6)*	*26.7 (24.5–29.4)*	*< 0.001*	*29.4 (26.3–32.6)*	*26.6 (24.2–29.7)*	*< 0.001*	*28.4 (25.3–32.2)*	*25.9 (23.5–28.9)*	*< 0.001*	*28.7 (26.0-31.8)*	*26.8 (24.5–29.5)*	*< 0.001*
***COPD***,*** n (%)***	*n/a*	*n/a*	*n/a*	*1*,*219 (11.6)*	*2*,*401 (8.4)*	*< 0.001*	*215 (15.0)*	*769 (11.7)*	*< 0.001*	*719 (22.0)*	*1556 (18.2)*	*< 0.001*	*215 (15.8)*	*421 (11.9)*	*< 0.001*
***Prior card. pro.***,*** n (%)***	*n/a*	*n/a*	*n/a*	*184 (1.8)*	*437 (1.5)*	*0.049*	*129 (9.0)*	*514 (7.8)*	*0.14*	*713 (21.8)*	*1556 (18.2)*	*< 0.001*	*42 (3.1)*	*104 (3.0)*	*0.851*
***Multi vessel dis.***,*** n (%)***	*22*,*145 (57.9)*	*65*,*394* (47.0)	*< 0.001*	*9*,*526 (91.0)*	*25*,*714 (89.9)*	*< 0.001*	*n/a*	*n/a*	*n/a*	*n/a*	*n/a*	*n/a*	*886 (65.0)*	*2181 (61.9)*	*0.016*
***Prior MI***,*** n (%)***	*11*,*263 (29.5)*	*26*,*909 (19.3)*	*< 0.001*	*3*,*592 (34.3)*	*10*,*251 (35.8)*	*0.003*	*n/a*	*n/a*	*n/a*	*n/a*	*n/a*	*n/a*	*182 (13.3)*	*398 (11.3)*	*0.043*
***Urgent procedure***,*** n (%)***	*n/a*	*n/a*	*n/a*	*4*,*522 (43.2)*	*12*,*608 (44.1)*	*0.112*	*191 (13.4)*	*856 (13.1)*	*0.774*	*n/a*	*n/a*	*n/a*	*343 (25.2)*	*845 (23.9)*	*0.344*
***Log. EuroSCORE I***,*** median (IQR)***	*n/a*	*n/a*	*n/a*	*3.2 (1.8–5.9)*	*2.7 (1.5-5.0)*	*< 0.001*	*5.14 (3.4-8.0)*	*4.5 (2.7–7.1)*	*< 0.001*	*12.04 (7.98–19.25)*	*11.72 (8.23-18.0)*	*0.112*	*6. (3.9–10.1)*	*5.7 (3.7–9.2)*	*0.004*
***Log. EuroSCORE II***,*** median (IQR)***	*n/a*	*n/a*	*n/a*	*1.7 (1.1–2.9*	*1.3 (0.9–2.1)*	*< 0.001*	*1.5 (1.0-2.4)*	*1.3 (0.9-2.0)*	*< 0.001*	*3.9 (2.3–6.9)*	*3.3 (2.0-5.5)*	*< 0.001*	*3.1 (2.0-5.3)*	*2.6 (1.8–4.2)*	*< 0.001*
***Active endocarditis***,*** n (%)***	*n/a*	*n/a*	*n/a*	*n/a*	*n/a*	*n/a*	*76 (5.3)*	*382 (5.8)*	*0.448*	*n/a*	*n/a*	*n/a*	*19 (1.4)*	*45 (1.3)*	*0.746*
***Prior CVA***,*** n (%)***	*n/a*	*n/a*	*n/a*	*711 (6.8)*	*1*,*224 (4.3)*	*< 0.001*	*96 (6.6)*	*344 (5.2)*	*0.034*	*381 (11.6)*	*841 (9.8)*	*0.004*	*111 (8.2)*	*241 (6.9)*	*0.082*
***CTO***,*** n (%)***	*2*,*451 (6.4)*	*7*,*004 (5.0)*	*< 0.001*	*n/a*	*n/a*	*n/a*	*n/a*	*n/a*	*n/a*	*n/a*	*n/a*	*n/a*	*n/a*	*n/a*	*n/a*
***Shock***,*** n (%)***	*903 (2.3)*	*3*,*890 (2.8)*	*< 0.001*	*n/a*	*n/a*	*n/a*	*n/a*	*n/a*	*n/a*	*n/a*	*n/a*	*n/a*	*n/a*	*n/a*	*n/a*
***OHCA***,*** n (%)***	*919 (2.3)*	*5*,*335 (3.8)*	*< 0.001*	*n/a*	*n/a*	*n/a*	*n/a*	*n/a*	*n/a*	*n/a*	*n/a*	*n/a*	*n/a*	*n/a*	*n/a*
***Prior CABG***,*** n (%)***	*5*,*864 (15.3)*	*10*,*818 (7.8)*	*< 0.001*	*n/a*	*n/a*	*n/a*	*n/a*	*n/a*	*n/a*	*n/a*	*n/a*	*n/a*	*n/a*	*n/a*	*n/a*
***PCI (STEMI)***,*** n (%)***	*7*,*770 (20.3)*	*45*,*529 (32.7)*	*< 0.001*	*n/a*	*n/a*	*n/a*	*n/a*	*n/a*	*n/a*	*n/a*	*n/a*	*n/a*	*n/a*	*n/a*	*n/a*
***NYHA-class IV***,*** n (%)***	*n/a*	*n/a*	*n/a*	*n/a*	*n/a*	*n/a*	*n/a*	*n/a*	*n/a*	*247 (7.6)*	*416 (5.0)*	*< 0.001*	*n/a*	*n/a*	*n/a*

*n/a = not applicable (not part of NHR indicators),* eGFR = Estimated Glomerular Filtration Rate*,* LVEF = Left ventricular ejection fraction*,* BMI = body mass index*,* COPD = chronic obstructive pulmonary disease*,* Prior. Card. Pro. = prior cardiac procedure*,* Multi vessel dis. = multi vessel disease*,* prior MI = prior myocardial infarction*,* log. EuroSCORE I = logistic EuroSCORE I*,* log. EuroSCORE II = logistic EuroSCORE II*,* prior CVA = cerebrovascular accident*, *OHCA = out of hospital arrest. An overview of the available baseline characteristics per procedure is shown in Table *1*of the Supplementary materials*

Table 2 shows descriptive and univariable analyses of outcome variables between patients with diabetes and patients without diabetes for each cardiac procedure. For PCI, there is a significant difference between patients with and without diabetes in 30-day mortality, 1-year mortality, myocardial infarction within 30 days after the procedure and TVR within 1 year after the procedure (*p* <.001). For CABG, 120-day mortality, 1-year mortality, CVA, DSWI within 30 days (*p* <.001) and coronary reintervention during follow-up (*p* <.001) are statistically significant. In the AVR cohort, there is a statistically significant difference between 120-day mortality (*p* =.003), 1-year mortality (*p* =.031), and DSWI within 30 days (*p* <.001). Concerning TAVI, 120-day mortality (*p* =.035), 1-year mortality (*p* <.001) and implantation of a new permanent pacemaker within 30 days post-procedure (*p* =.014) are statistically different between the two groups. Last, CABG + AVR has a statistically significant difference between patients with diabetes and patients without diabetes regarding 120-day mortality (*p* <.001), 1-year mortality (*p* =.023) and DSWI within 30 days (*p* =.030).

**Table 2:** Univariable analyses of outcome variables between diabetics and non-diabetics for each cardiac procedure

	Coronary artery disease (CAD)						Aortic Valve Disease (AVD)						Combined CAD and AVD		
	PCI *n* = 177,556			CABG *N* = 39,069			AVR *N* = 8,028			TAVI *N* = 11,819			CABG + AVR *N* = 4,888		
	DM	No DM	*p*-value	DM	No DM	*p*-value	DM	No DM	*p*-value.	DM	No DM	*p*-value.	DM	No DM	*p-value.*
***Mortality***															
***Proc. mortality (3-days)***,*** n (%)***	*n/a*	*n/a*	*n/a*	*n/a*	*n/a*	*n/a*	*n/a*	*n/a*	*n/a*	*26 (0.8)*	*89 (1.0)*	*0.217*	*n/a*	*n/a*	*n/a*
***30-day mortality***,*** n (%)***	*1*,*332 (3.5)*	*3*,*385 (2.5)*	*< 0.001*	*n/a*	*n/a*	*n/a*	*n/a*	*n/a*	*n/a*	*102 (3.1)*	*244 (2.9)*	*0.458*	*n/a*	*n/a*	*n/a*
***120-day mortality***,*** n (%)***	*n/a*	*n/a*	*n/a*	*264 (2.6)*	*438 (1.6)*	*< 0.001*	*43 (3.1)*	*117 (1.8)*	*0*,*003*	*192 (6.1)*	*420 (5.1)*	*0*,*035*	*82 (6.2)*	*134 (3.9)*	*< 0.001*
***1-year mortality (2015–2019)***,*** n (%)***	*2*,*745 (8.5)*	*5*,*782 (4.9)*	*< 0.001*	*334 (3.8)*	*546 (2.3)*	*< 0.001*	*52 (4.2)*	*165 (3.0)*	*0*,*031*	*334 (12.9)*	*698 (10.2)*	*< 0.001*	*83 (7.2)*	*161 (5.3)*	*0.023*
***Complications***															
***CVA***,*** during admission n (%)***	*n/a*	*n/a*	*n/a*	*95 (09)*	*169 (0.6)*	*< 0.001*	*15 (1.1)*	*57 (0.9)*	*0.547*	*61 (2.0)*	*157 (1.9)*	*0.884*	*26 (1.9)*	*67 (1.9)*	*1.000*
***Re-exploration < 30 days***,*** n (%)***	*n/a*	*n/a*	*n/a*	*50 (0.5)*	*129 (0.7)*	*0.334*	*79 (5.7)*	*361 (5.7)*	*0.944*	*n/a*	*n/a*	*n/a*	*115 (8.8)*	*263 (7.7)*	*0.245*
***DSWI < 30 days***,*** n (%)***	*n/a*	*n/a*	*n/a*	*171 (1.7)*	*198 (0.7)*	*< 0.001*	*14 (1.0)*	*20 (0.3)*	*< 0.001*	*n/a*	*n/a*	*n/a*	*20 (1.5)*	*26 (0.8)*	*0.030*
***PM < 30 days***,*** n (%)***	*n/a*	*n/a*	*n/a*	*n/a*	*n/a*	*n/a*	*33 (3.7)*	*168 (4.1)*	*0.613*	*371 (11.6)*	*842 (10.1)*	*0*,*014*	*22 (2.6)*	*81 (3.6)*	*0.178*
***Maj. vasc. compl. < 30 days***,*** n (%)***	*n/a*	*n/a*	*n/a*	*n/a*	*n/a*	*n/a*	*n/a*	*n/a*	*n/a*	*73 (2.6)*	*243 (3.2)*	*0.097*	*n/a*	*n/a*	*n/a*
***MI < 30 days***,*** n (%)***	*219 (0.9)*	*568 (0.7)*	*< 0.001*	*n/a*	*n/a*	*n/a*	*n/a*	*n/a*	*n/a*	*n/a*	*n/a*	*n/a*	*n/a*	*n/a*	*n/a*
***Urgent CABG < 1 day***,*** n (%)***	*69 (0.2)*	*262 (0.2)*	*0.323*	*n/a*	*n/a*	*n/a*	*n/a*	*n/a*	*n/a*	*n/a*	*n/a*	*n/a*	*n/a*	*n/a*	*n/a*
***TVR < 1 year (2015–2019)***,*** n (%)***	*2*,*109 (7.3)*	*5*,*678 (5.3)*	*< 0.001*	*n/a*	*n/a*	*n/a*	*n/a*	*n/a*	*n/a*	*n/a*	*n/a*	*n/a*	*n/a*	*n/a*	*n/a*

*n/a = not applicable (not part of NHR indicators),* proc. Mortality (3-days) = procedural mortality within 3 days*,* PM < 30-days = implantation of new permanent pacemaker within 30 days*,* maj. vasc. compl < 30-days = major vascular complication within 30 days*,* MI < 30 days = myocardial infarction within 30 days*,* TVR < 1 year = Target Vessel Revascularization within 1 year. An overview of the available baseline characteristics per procedure is shown in*Table 1*of the Supplementary materials*

The results of the logistic regression analyses with diabetes as an independent variable are shown in Table 3. Diabetes mellitus is a statistically significant variable in the PCI-cohort for 30-day mortality (*p* <.001; OR = 1.668), 1-year mortality (p = < 0.001; OR = 1.682), myocardial infarction within 30-days (*p* <.001; OR = 1.431) and TVR within 1 year (*p* <.001; OR = 1.307). In the CABG-cohort, diabetes mellitus is significantly associated with the following outcome measures: 120-day mortality (*p* <.001; OR = 1.421), 1-year mortality (*p* <.001; OR = 1.494), CVA (*p* =.015; OR = 1.371) and DWSI within 30-days post-procedure (*p* <.001; OR = 2.203). Concerning TAVI, 1-year mortality (*p* <.010; OR = 1.122) and implantation of a new permanent pacemaker within 30 days post-procedure (*p* =.007; OR = 1.200) are significantly associated with diabetes. Lastly, for AVR, diabetes is a statistically significant variable for 120-day mortality (*p* =.030, OR = 1.493), DWSI within 30-days (*p* =.003; OR = 2.873) and for CABG + AVR the variables 120-day mortality (*p* =.005; OR = 1.520) and DSWI within 30-days (*p* =.022; 1.996). Results of the additional sensitivity analyses and propensity score matching can be found in Tables 3, 4, 5 and 6 in the Supplementary materials; all analyses led to similar findings.

**Table 3:** Results of the multivariate logistic regression analyses with correlation between diabetes mellitus and all early outcomes

Outcome measure	Coronary artery disease (CAD)				Aortic valve disease (AVD)				Combined CAD and AVD	
	PCI *n* = 177,556		CABG *N* = 39,069		AVR *N* = 8,028		TAVI *N* = 11,819		CABG + AVR *N* = 4,888	
	OR (95% CI)	*p*-value	OR (95% CI)	*p*-value	OR (95% CI)	*p*-value	OR (95% CI)	*p*-value	OR (95% CI)	*p*-value
***Mortality***										
***Proc. mortality (3-days)***	n/a	n/a	n/a	n/a	n/a	n/a	0.72 (0.46-1.13)	0.15	n/a	n/a
***30-day mortality***	1.68 (1.56–1.82)	< 0.001	n/a	n/a	n/a	n/a	1.03 (0.81-1.31)	0.81	n/a	n/a
***120-day mortality***	n/a	n/a	1.35 (1.15–1.58)	< 0.001	1.50 (1.04–2.15)	0.03	1.13 (0.95-1.36)	0.18	1.42 (1.05–1.90)	0.02
***1-year mortality (2015–2019)***	1.68 (1.6–1.77)	< 0.001	1.43 (1.24–1.65)	< 0.001	1.22 (0.88-1.69)	0.23	1.21 (1.05–1.4)	0.01	1.21 (0.91-1.61)	0.19
***Complications***										
***CVA during admission***	n/a	n/a	1.32 (1.02–1.7)	0.04	1.15 (0.65-2.04)	0.64	1.05 (0.77-1.42)	0.77	1.02 (0.65-1.62)	0.93
***Re-exploration < 30 days***	n/a	n/a	0.92 (0.82-1.03)	0.15	0.99 (0.77-1.28)	0.94	n/a	n/a	1.14 (0.91-1.44)	0.26
***DSWI < 30 days***	n/a	n/a	2.18 (1.77–2.68)	< 0.001	2.86 (1.43–5.69)	0.003	n/a	n/a	1.92 (1.06–3.48)	0.03
***PM < 30 days***	n/a	n/a	n/a	n/a	0.88 (0.60-1.29)	0.51	1.20 (1.05–1.37)	0.01	0.68 (0.42 − 1.10)	0.11
***Maj. vasc. compl. < 30 days***	n/a	n/a	n/a	n/a	n/a	n/a	0.82 (0.63-1.08)	0.16	n/a	n/a
***MI < 30 days***	1.44 (1.24–1.67)	< 0.001	n/a	n/a	n/a	n/a	n/a	n/a	n/a	n/a
***Urgent CABG < 1 day***	0.94 (0.72-1.22)	0.64	n/a	n/a	n/a	n/a	n/a	n/a	n/a	n/a
***TVR < 1 year (2015–2019)***	1.30 (1.23–1.37)	< 0.001	n/a	n/a	n/a	n/a	n/a	n/a	n/a	n/a

* n/a = not applicable (not part of NHR indicators),* proc. Mortality (3-days) = procedural mortality within 3 days*,* PM < 30-days = implantation of new permanent pacemaker within 30 days*,* maj. vasc. compl < 30-days = major vascular complication within 30 days*,* MI < 30 days = myocardial infarction within 30 days*,* TVR < 1 year = Target Vessel Revascularization within 1 year. An overview of the available baseline characteristics per procedure is shown in Table *1*of the Supplementary materials*

**Fig. 1:**
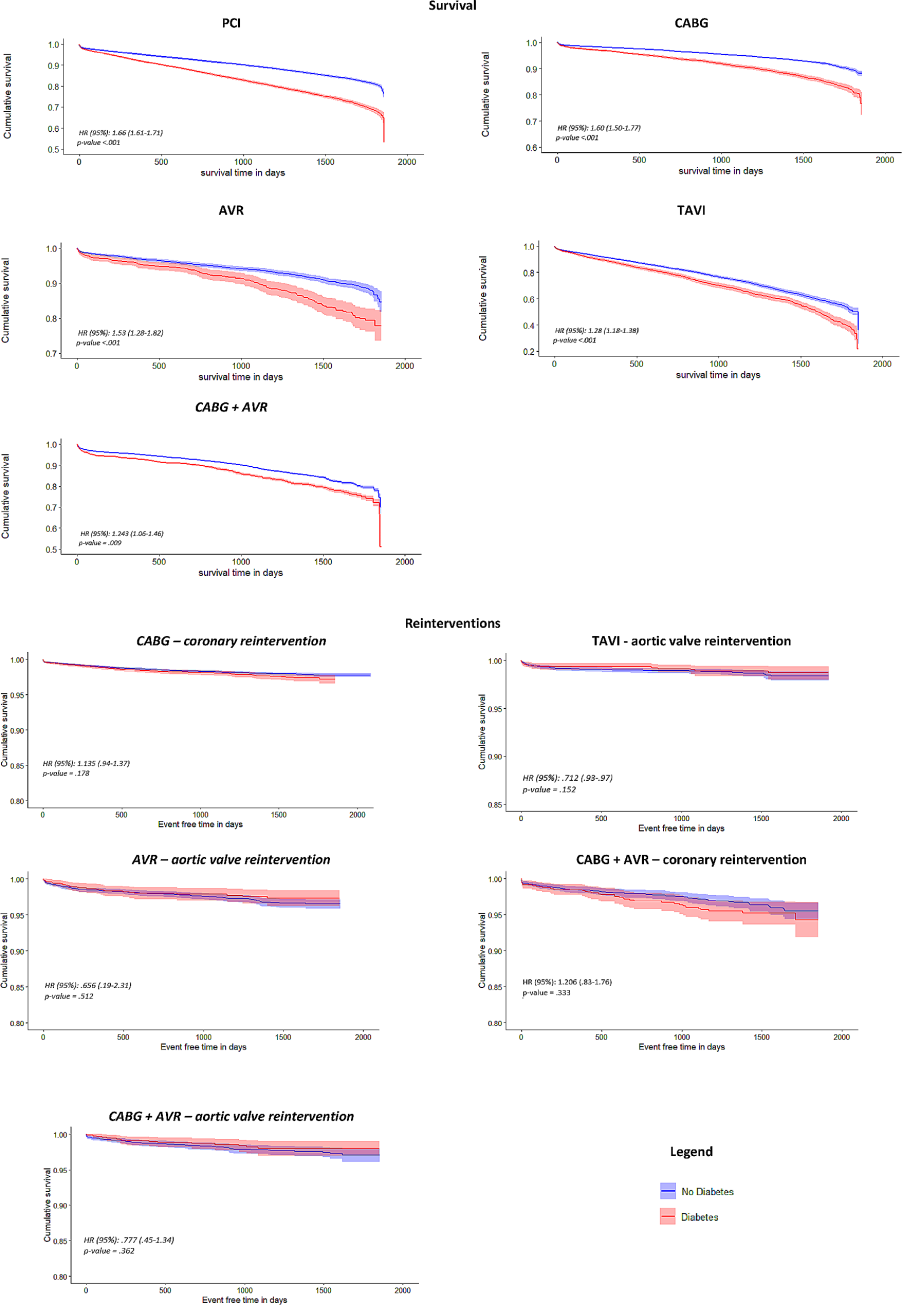
Cox regression curves per intervention of the study regarding survival and reinterventions. Blue lines; no diabetes mellitus, Red lines; diabetes mellitus

Last, Fig. 1 below shows the cox regression survival curves for each intervention. Survival time in days for patients living with diabetes and patients living without diabetes is plotted over a follow-up period of five years, adjusted for all baseline characteristics. All figures show patients with diabetes have lower survival rates compared to patients without diabetes.

## Discussion

The current study provides the latest report on the relationship between diabetes mellitus and clinical outcomes after different invasive cardiac interventions in the Netherlands. This study uses recent, real-world data from multiple years (2015–2020) from a nationwide registry covering almost the entire Dutch CVD population that underwent invasive cardiac interventions. It thus forms a representative sample of all cardiac procedures on a nearby nationwide level. Results show that people with diabetes have statistically significantly worse outcomes in terms of mortality, complications and reinterventions compared to people without diabetes.

From a pathophysiologic point of view, these findings could be expected as DM patients share similar risk factors for CAD (such as hypercholesterolemia, obesity, etc.) and DM itself is associated with endothelial damage as a result of glucose-related tissue toxicity and circulating inflammatory cytokines [29]. Quantitative histologic analysis of aortic valves after explantation for AVR shows worse calcifications in DM patients and a recent animal study points in the direction of faster progression to calcification of the aortic valve in DM-hyperglycemic hamsters [30]. The association between DM and AVD is not well understood but given that calcification of the aortic valve and DM both are related to inflammatory pathways, an association is likely [31].

Historically, treatment of DM focused on drug therapy, aimed to control glycemic control, hypertension and cholesterol levels. In recent years, a paradigm shift toward a more patient-centred approach addressing lifestyle-related risks such as increasing physical activity levels, adopting a healthy and varied diet to control weight, and smoking cessation [9, 16, 32, 33]. Since 2019, the European Society of Cardiology included Sodium-glucose co-transporter 2 (SGLT2-inhibitors) in the ESC Clinical Practice guidelines, based on new evidence that SGLT2-inhibitors benefit patients with cardiovascular diseases and reduce the risk of cardiovascular mortality [34, 35]. Nevertheless, the present study shows that differences between patients with diabetes and patients without diabetes still exist; patients with diabetes have worse outcomes and mortality statistics than patients without diabetes. As shown in our study, patients with diabetes have higher odds of mortality compared to patients without diabetes. People with diabetes show higher healthcare utilization and higher healthcare costs compared to people without diabetes, whereas the prevalence of DM is deemed to strongly incline in the upcoming decades [3, 9, 10, 16].

With regard to CAD interventions, the results align with two recent meta-analyses by Head et al. 2018 [36] and Zhou et al. 2019 [14]and two cohort studies by Rogan et al. 2018 [37] and Raza et al. 2015 [3]. These studies, covering both randomized controlled trials (RCT) and observational studies for a period ranging between 1972 and 2018, state people with diabetes have significantly worse outcomes concerning short-term mortality and 1-year mortality [3, 14, 36–38]. Concerning complications and reinterventions in literature; patients with diabetes have more strokes, bleeding complications, DSWI, MI and reinterventions [14, 39–42]. Our study confirms these results, although it is worth noting that our study uses more recent data, has a higher number of total interventions and the data exist of observational real-world data.

Concerning aortic valve interventions, two studies Besch., 2019 [43], an observational study of 18,154 patients, and Mendez-Bailion et al., 2019 [44], a randomized controlled trial (RCT), show no statistically significant difference between patients with diabetes and patients without diabetes in short-term and long-term mortality as well as post-procedural complications. The RCT studies of both Abramowitz [15] and Layrer [45] reported no significant difference between patients with diabetes and patients without diabetes regarding short-term and long-term mortality as well as complications after TAVI. However, after correcting the data for baseline characteristics, diabetes was significantly associated with mortality. This partly contradicts our study, covering more recent years, more interventions and is an observational study instead of an RCT; we did not find an association between diabetes mellitus and 30-day mortality, but we did find a negative association in relation to 1-year mortality and pacemaker implantation. Results specifically in relation to AVR are contradicting some previous publications; to our knowledge, there is no consensus in the literature on the difference between patients with diabetes and patients without diabetes in terms of mortality, complications or reinterventions. A recent systematic review by Banovic et al., 2019 [46]reports that results on short-term and long-term mortality and complications differ per study while other studies, such as Ram et al., 2019 [13], an observational study covering 1,053 patients, do not report differences between patients with diabetes and patients without diabetes. Our study, which includes recent data on more AVR patients, does show a significant difference in terms of 120-day mortality and DSWI within 30 days when correcting for baseline characteristics.

Over the years, mortality in cardiovascular diseases is decreasing for both patients with and without diabetes [9]. The systematic review of Htay et al., 2019 [9] found the gap between patients with diabetes and patients without diabetes to decrease; mortality among diabetics is declining at a higher rate than among non-diabetics. The study states developments in the treatment of diabetes and diabetic complications, together with improved management of individual risk factors, are the main influences for this decline [9]. In general, according to the studies mentioned above and the results of this study, people with diabetes have statistically significantly worse outcomes in terms of mortality, complications and reinterventions compared to people without diabetes. To prevent a rapid incline in disease burden and healthcare consumption, it is key to optimize healthcare protocols for this target group and increase focus on prevention. A possible solution is to create and optimize regional collaboration between healthcare providers to align healthcare pathways [47].

### Strengths and limitations

One of the strengths of this study is its size; in total 214,360 surgical and percutaneous cardiac procedures were included in a period of six years. It uses observational, real-world data from populations in daily practice instead of data received from included patients in an RCT. Hence, patients from almost the entire Dutch surgical and percutaneous cardiovascular disease population are included, which ensures a recent and proportionate representation. However, this dataset can also be seen as a limitation, since observational retrospective data have potential bias; arguably, not all relevant risk factors have been included in the dataset. In this study, a standard set of variables, as selected by medical experts in the registration committees of the NHR, was used. This selection of the most relevant outcomes and baseline characteristics is based on a published methodology. However, as this is a limited set it possibly excludes some baseline characteristics that might also influence outcomes (e.g. TAVI prior to PCI, SYNTAX score and STS PROM-scale). Also, relevant biomedical parameters, such as perioperative and postoperative blood plasma glucose levels, and data on diabetic medication as well as on antiplatelet treatment, are not collected in the NHR which made it impossible to correct for these variables in the multivariate analysis. This can potentially affect the reported association. For future research, it is recommended to include more variables if possible. Furthermore, quality of life is not included in these analyses due to the fact that the majority of hospitals have insufficiently collected these variables. Hence, a recommendation for future research is to study the difference between patients with diabetes and patients without diabetes after different invasive cardiac interventions. Additionally, due to multiplicity, there is a potential for an increased risk of a type I error. Last, although differences over time were studied, showing no statistical differences, the impact of the COVID-19 pandemic on the study results of 2020 is unknown. This should be included in future research, as SARS-CoV-2 could be associated with a higher risk of in-hospital mortality [48].

This study has shown that real-world, clinical outcomes after cardiac procedures still differ between patients with diabetes and patients without diabetes. It is vital to critically assess existing care pathways and protocols targeted to patients with diabetes to minimize these differences.

### Electronic supplementary material

Below is the link to the electronic supplementary material.

Supplementary Material 1

Supplementary Material 2

Supplementary Material 3

Supplementary Material 4

Supplementary Material 5

Supplementary Material 6

Supplementary Material 7

Supplementary Material 8

Supplementary Material 9

## Data Availability

The data underlying this article were provided by the Netherlands Heart Registration (NHR) by permission. A request can be submitted to NHR to access these data.

## References

[1] McGurnaghan S, Blackbourn LAK, Mocevic E, Haagen Panton U, McCrimmon RJ, Sattar N, et al. Cardiovascular disease prevalence and risk factor prevalence in type 2 diabetes: a contemporary analysis. Diabet Med. 2019;36(6):718–25. 30246473 10.1111/dme.13825 PMC6585697

[2] Einarson TR, Acs A, Ludwig C, Panton UH. Prevalence of cardiovascular disease in type 2 diabetes: a systematic literature review of scientific evidence from across the world in 2007–2017. Cardiovasc Diabetol. 2018;17(1):83. 29884191 10.1186/s12933-018-0728-6 PMC5994068

[3] Raza S, Sabik JF, Ainkaran P, Blackstone EH. Coronary artery bypass grafting in diabetics: a growing health care cost crisis. J Thorac Cardiovasc Surg. 2015;150(2):304–e3122. 26027913 10.1016/j.jtcvs.2015.03.041 PMC5120545

[4] Dunstan DW, Zimmet PZ, Welborn TA, de Courten MP, Cameron AJ, Sicree RA, et al. The rising prevalence of diabetes and impaired glucose tolerance. Diabetes Care. 2002;25(5):829–34. 11978676 10.2337/diacare.25.5.829

[5] Spallone V, Ziegler D, Freeman R, Bernardi L, Frontoni S, Pop-Busui R, et al. Cardiovascular autonomic neuropathy in diabetes: clinical impact, assessment, diagnosis, and management. Diabetes Metab Res Rev. 2011;27(7):639–53. 21695768 10.1002/dmrr.1239

[6] Hu FB. Globalization of diabetes. Diabetes Care. 2011;34(6):1249–57. 21617109 10.2337/dc11-0442 PMC3114340

[7] The Diabetes Control and complications trial (DCCT)/Epidemiology of Diabetes Interventions and Complications (EDIC) Study Research Group. Intensive Diabetes Treatment and Cardiovascular outcomes in Type 1 diabetes: the DCCT/EDIC Study 30-Year follow-up. Diabetes Care. 2016;39(5):686–93. 26861924 10.2337/dc15-1990 PMC4839174

[8] Preis SR, Pencina MJ, Hwang SJ, D’Agostino RB, Savage PJ, Levy D, et al. Trends in cardiovascular disease risk factors in individuals with and without diabetes mellitus in the Framingham Heart Study. Circulation. 2009;120(3):212–20. 19581493 10.1161/CIRCULATIONAHA.108.846519 PMC2789428

[9] Htay T, Soe K, Lopez-Perez A, Doan AH, Romagosa MA, Aung K. Mortality and Cardiovascular Disease in Type 1 and type 2 diabetes. Curr Cardiol Rep. 2019;21(6):45. 31011838 10.1007/s11886-019-1133-9

[10] Struijs JN, Baan CA, Schellevis FG, Westert GP, van den Bos GA. Comorbidity in patients with diabetes mellitus: impact on medical health care utilization. BMC Health Serv Res. 2006;6(1):84. 16820048 10.1186/1472-6963-6-84 PMC1534031

[11] Leon BM. Diabetes and cardiovascular disease: Epidemiology, biological mechanisms, treatment recommendations and future research. World J Diabetes. 2015;6(13):1246. 26468341 10.4239/wjd.v6.i13.1246 PMC4600176

[12] Sowers JR, Epstein M, Frohlich ED. Diabetes, hypertension, and Cardiovascular Disease. Hypertension. 2001;37(4):1053–9. 11304502 10.1161/01.HYP.37.4.1053

[13] Ram E, Kogan A, Levin S, Fisman EZ, Tenenbaum A, Raanani E, et al. Type 2 diabetes mellitus increases long-term mortality risk after isolated surgical aortic valve replacement. Cardiovasc Diabetol. 2019;18(1):31. 30876424 10.1186/s12933-019-0836-y PMC6419403

[14] Zhuo X, Zhang C, Feng J, Ouyang S, Niu P, Dai Z. In-hospital, short-term and long-term adverse clinical outcomes observed in patients with type 2 diabetes mellitus vs non-diabetes mellitus following percutaneous coronary intervention. Medicine. 2019;98(8):e14669. 30813214 10.1097/MD.0000000000014669 PMC6408074

[15] Abramowitz Y, Jilaihawi H, Chakravarty T, Mangat G, Maeno Y, Kazuno Y, et al. Impact of diabetes Mellitus on outcomes after Transcatheter aortic valve implantation. Am J Cardiol. 2016;117(10):1636–42. 27015888 10.1016/j.amjcard.2016.02.040

[16] Marín-Peñalver JJ, Martín-Timón I, Sevillano-Collantes C, del Cañizo-Gómez FJ. Update on the treatment of type 2 diabetes mellitus. World J Diabetes. 2016;7(17):354–95. 27660695 10.4239/wjd.v7.i17.354 PMC5027002

[17] Ghosh-Swaby OR, Goodman SG, Leiter LA, Cheng A, Connelly KA, Fitchett D, et al. Glucose-lowering drugs or strategies, atherosclerotic cardiovascular events, and heart failure in people with or at risk of type 2 diabetes: an updated systematic review and meta-analysis of randomised cardiovascular outcome trials. Lancet Diabetes Endocrinol. 2020;8(5):418–35. 32333878 10.1016/S2213-8587(20)30038-3

[18] Timmermans MJC, Houterman S, Daeter ED, Danse PW, Li WW, Lipsic E et al. Using real-world data to monitor and improve quality of care in coronary artery disease: results from the Netherlands Heart Registration. Neth Heart J. 2022. 10.1007/s12471-022-01672-0 PMC8988537 35389133

[19] Daeter EJ, Timmermans MJC, Hirsch A, Lipsic E, Houterman S, van Veghel D, et al. Defining and measuring a Standard Set of patient-relevant Outcomes in Coronary Artery Disease. Am J Cardiol. 2018;121(12):1477–88. 29776654 10.1016/j.amjcard.2018.02.037

[20] Houterman S, van Dullemen A, Versteegh M, Aengevaeren W, Danse P, Brinkman E et al. Data quality and auditing within the Netherlands Heart Registration: using the PCI registry as an example. Neth Heart J. 2023. 10.1007/s12471-022-01752-1 PMC10444924 36645544

[21] Netherlands Heart Registration. Handboek Nederlandse Hart Registratie 2021. Utrecht; 2021 Sep.

[22] American Diabetes Association. Diagnosis and classification of diabetes Mellitus. Diabetes Care. 2013;36(Supplement1):S67–74. 23264425 10.2337/dc13-S067 PMC3537273

[23] The Task Force on diabetes. Pre diabetes, and cardiovascular diseases of the ES of C (ESC) and developed in collaboration with the EA for the S of D (EASD). ESC guidelines on diabetes, pre-diabetes, and cardiovascular diseases developed in collaboration with the EASD– summary. Diab Vasc Dis Res. 2014;11(3):133–73. 24800783 10.1177/1479164114525548

[24] Thygesen K, Alpert JS, Jaffe AS, Simoons ML, Chaitman BR, White HD, et al. Third universal definition of myocardial infarction. Eur Heart J. 2012;33(20):2551–67. 22922414 10.1093/eurheartj/ehs184

[25] Cannon CP, Battler A, Brindis RG, Cox JL, Ellis SG, Every NR, et al. American College of Cardiology key data elements and definitions for measuring the clinical management and outcomes of patients with acute coronary syndromes. J Am Coll Cardiol. 2001;38(7):2114–30. 11738323 10.1016/S0735-1097(01)01702-8

[26] Sacco RL, Kasner SE, Broderick JP, Caplan LR, Connors JJB, Culebras A, et al. An updated definition of stroke for the 21st century: a statement for healthcare professionals from the American Heart Association/American Stroke Association. Stroke. 2013;44(7):2064–89. 23652265 10.1161/STR.0b013e318296aeca PMC11078537

[27] Rippe RCA, den Heijer M, le Cessie S. Imputation of missing data. Ned Tijdschr Geneeskd. 2013;157(18):A5539. 23635501

[28] Jakobsen JC, Gluud C, Wetterslev J, Winkel P. When and how should multiple imputation be used for handling missing data in randomised clinical trials– a practical guide with flowcharts. BMC Med Res Methodol. 2017;17(1):162. 29207961 10.1186/s12874-017-0442-1 PMC5717805

[29] Rodriguez-Araujo G, Nakagami H. Pathophysiology of cardiovascular disease in diabetes mellitus. Cardiovasc Endocrinol Metab. 2018;7(1):4–9. 31646271 10.1097/XCE.0000000000000141 PMC6739883

[30] Mosch J, Gleissner CA, Body S, Aikawa E. Histopathological assessment of calcification and inflammation of calcific aortic valves from patients with and without diabetes mellitus. Histol Histopathol. 2017;32(3):293–306. 27353274 10.14670/HH-11-797 PMC5199639

[31] Manduteanu I, Simionescu D, Simionescu A, Simionescu M. Aortic valve disease in diabetes: molecular mechanisms and novel therapies. J Cell Mol Med. 2021;25(20):9483–95. 34561944 10.1111/jcmm.16937 PMC8505854

[32] Cosentino F, Grant PJ, Aboyans V, Bailey CJ, Ceriello A, Delgado V, et al. 2019 ESC guidelines on diabetes, pre-diabetes, and cardiovascular diseases developed in collaboration with the EASD. Eur Heart J. 2020;41(2):255–323. 31497854 10.1093/eurheartj/ehz486

[33] Inzucchi SE, Bergenstal RM, Buse JB, Diamant M, Ferrannini E, Nauck M, et al. Management of hyperglycemia in type 2 diabetes, 2015: a patient-centered Approach: update to a position Statement of the American Diabetes Association and the European Association for the study of diabetes. Diabetes Care. 2015;38(1):140–9. 25538310 10.2337/dc14-2441

[34] McGuire DK, Shih WJ, Cosentino F, Charbonnel B, Cherney DZI, Dagogo-Jack S, et al. Association of SGLT2 inhibitors with Cardiovascular and kidney outcomes in patients with type 2 diabetes. JAMA Cardiol. 2021;6(2):148. 33031522 10.1001/jamacardio.2020.4511 PMC7542529

[35] Zelniker TA, Wiviott SD, Raz I, Im K, Goodrich EL, Bonaca MP, et al. SGLT2 inhibitors for primary and secondary prevention of cardiovascular and renal outcomes in type 2 diabetes: a systematic review and meta-analysis of cardiovascular outcome trials. Lancet. 2019;393(10166):31–9. 30424892 10.1016/S0140-6736(18)32590-X

[36] Head SJ, Milojevic M, Daemen J, Ahn JM, Boersma E, Christiansen EH, et al. Mortality after coronary artery bypass grafting versus percutaneous coronary intervention with stenting for coronary artery disease: a pooled analysis of individual patient data. Lancet. 2018;391(10124):939–48. 29478841 10.1016/S0140-6736(18)30423-9

[37] Kogan A, Ram E, Levin S, Fisman EZ, Tenenbaum A, Raanani E, et al. Impact of type 2 diabetes mellitus on short- and long-term mortality after coronary artery bypass surgery. Cardiovasc Diabetol. 2018;17(1):151. 30497472 10.1186/s12933-018-0796-7 PMC6264047

[38] Chichareon P, Modolo R, Kogame N, Takahashi K, Chang CC, Tomaniak M, et al. Association of diabetes with outcomes in patients undergoing contemporary percutaneous coronary intervention: pre-specified subgroup analysis from the randomized GLOBAL LEADERS study. Atherosclerosis. 2020;295:45–53. 32006758 10.1016/j.atherosclerosis.2020.01.002

[39] Agarwal K, Ganai J. Impact of diabetes on the patients undergoing coronary artery bypass grafting. SSRN Electron J. 2022.

[40] Badour SA, Dimitrova KR, Kanei Y, Tranbaugh RF, Hajjar MM, Kabour A, et al. First and second generation DESs reduce diabetes adverse effect on mortality and re-intervention in multivessel coronary disease: 9-Year analysis. Cardiovasc Revascularization Med. 2017;18(4):265–73. 10.1016/j.carrev.2017.01.012 28314676

[41] Popov T, Dejanovic J, Petrovic M, Srdanovic I, Tadic S, Vulin A et al. P5545Predictors of 10-year mortality and re-intervention in patients with multivessel coronary disease, reduced systolic left ventricular function, after complete revascularization by PCI or CABG. Eur Heart J. 2019;40(Supplement_1).

[42] Haqzad Y, Hobkirk J, Ariyaratnam P, Chaudhry M, Carroll S, Loubani M. Outcomes following coronary artery bypass surgery in diabetic treatment sub-groups. A propensity matched analysis of >7000 patients over 18 years. Asian Cardiovasc Thorac Ann. 2022;30(2):131–40. 33730864 10.1177/0218492321999551

[43] Besch G, Pili-Floury S, Morel C, Gilard M, Flicoteaux G, Salomon du Mont L, et al. Impact of post-procedural glycemic variability on cardiovascular morbidity and mortality after transcatheter aortic valve implantation: a post hoc cohort analysis. Cardiovasc Diabetol. 2019;18(1):27. 30857532 10.1186/s12933-019-0831-3 PMC6410509

[44] Mendez-Bailon M, Lorenzo-Villalba N, Muñoz-Rivas N, de Miguel-Yanes JM, de Miguel-Diez J, Comín-Colet J, et al. Transcatheter aortic valve implantation and surgical aortic valve replacement among hospitalized patients with and without type 2 diabetes mellitus in Spain (2014–2015). Cardiovasc Diabetol. 2017;16(1):144. 29121921 10.1186/s12933-017-0631-6 PMC5679322

[45] Lareyre F, Mialhe C, Bourlon F, Habib Y, Dommerc C, Raffort J. Diabetes mellitus is not associated with worse vascular outcome following percutaneous transfemoral transcatheter aortic valve implantation. Acta Cardiol. 2019;74(6):480–6. 30642229 10.1080/00015385.2018.1522074

[46] Banovic M, Athithan L, McCann GP. Aortic stenosis and diabetes mellitus: an ominous combination. Diab Vasc Dis Res. 2019;16(4):310–23. 30623669 10.1177/1479164118820657

[47] Stork ADM, Oosterwerf-Suiker MM, Knippels M, Erdtsieck RJ, Kuijpers-Mannaerts L, van Lieshout D et al. Transmuraal formularium diabetes mellitus type 2 Zorggroepen 2022. 2022.

[48] De Luca G, Silverio A, Verdoia M, Siudak Z, Tokarek T, Kite TA, et al. Angiographic and clinical outcome of SARS-CoV-2 positive patients with ST-segment elevation myocardial infarction undergoing primary angioplasty: a collaborative, individual patient data meta-analysis of six registry-based studies. Eur J Intern Med. 2022;105:69–76. 35999094 10.1016/j.ejim.2022.08.021 PMC9385833

