# Supplementary material for: A high-volume study on the impact of diabetes mellitus on clinical outcomes after surgical and percutaneous cardiac interventions
Source: Cardiovasc Diabetol. 2024 Jul 18;23:260. doi: 10.1186/s12933-024-02356-2 (PMC11264856; doi:10.1186/s12933-024-02356-2)
Supplement: Supplementary file 1 — Supplementary Material 1 [file 12933_2024_2356_MOESM1_ESM.docx]

**Addendum**

The following physicians are members of the Cardiothoracic Surgery Registration Committee of the NHR. They represent the hospitals that have provided the CABG data for this study.

| S | Bramer | Amphia |
| --- | --- | --- |
| WJP | Van Boven | Amsterdam University Medical Centre, University of Amsterdam |
| ABA | Vonk | Amsterdam University Medical Centre, VU Medical Centre |
| BMJA | Koene | Catharina Hospital |
| JA | Bekkers | Erasmus Medical Centre |
| GJF | Hoohenkerk | Haga Hospital |
| ALP | Markou | Isala |
| A | De Weger | Leiden University Medical Centre |
| P | Segers | Maastricht University Medical Centre |
| F | Porta | Medical Centre Leeuwarden |
| RGH | Speekenbrink | Medical Spectrum Twente |
| W | Stooker | OLVG |
| WWL | Li | Radboud University Medical Centre |
| EJ | Daeter | St. Antonius Hospital |
| NP | Van der Kaaij | University Medical Centre of Utrecht |
| YL | Douglas | University Medical Centre Groningen |

The following physicians are members of the PCI Registration Committee of the NHR. They represent the hospitals that have provided the PCI data for this study.

| M | Scholte | Albert Schweitzer Hospital |
| --- | --- | --- |
| M | Meuwissen | Amphia |
| JP | Henriques | Amsterdam University Medical Centre, University of Amsterdam |
| KMJ | Marques | Amsterdam University Medical Centre, VU Medical Centre |
| T | Teeuwen | Catharina Hospital |
| H | Al Hashimi | Canisius Wilhelmina Hospital |
| M | Magro | Elisabeth-TweeSteden Hospital |
| J | Daemen | Erasmus Medical Centre |
| BJ | Sorgdrager | Haaglanden Medical Centre |
| CE | Schotborgh | Haga Hospital |
| V | Roolvink | Isala |
| J | Polad | Jeroen Bosch Hospital |
| I | Karalis | Leiden University Medical Centre |
| M | Van der Ent | Maasstad Hospital |
| AJW | Van ‘t Hof | Maastricht University Medical Centre |
| F | Spano | Meander Medical Centre |
| J | Brouwer | Medical Centre Leeuwarden |
| MG | Stoel | Medical Spectrum Twente |
| A | Dedic | Noordwest Hospital Group |
| G | Amoroso | OLVG |
| C | Camaro | Radboud University Medical Centre |
| PW | Danse | Rijnstate |
| JP | Van Kuijk | St. Antonius Hospital |
| EK | Arkenbout | Tergooi |
| WT | Ruifrok | Treant Zorggroep, Scheper Hospital |
| A | Kraaijeveld | University Medical Centre of Utrecht |
| E | Lipsic | University Medical Centre Groningen |
| S | Aydin | VieCuri Medical Centre |
| R | Erdem | ZorgSaam Hospital |
| AJW | Van ‘t Hof | Zuyderland Medical Centre |
