# Supplementary material for: A high-volume study on the impact of diabetes mellitus on clinical outcomes after surgical and percutaneous cardiac interventions
Source: Cardiovasc Diabetol. 2024 Jul 18;23:260. doi: 10.1186/s12933-024-02356-2 (PMC11264856; doi:10.1186/s12933-024-02356-2)
Supplement: Supplementary file 2 — Supplementary Material 2 [file 12933_2024_2356_MOESM2_ESM.docx]

***SUPPLEMENTARY Table 1. Included patient characteristics and outcome measures per cardiac procedure.***

| **Variable** | **PCI, *N (% complete)*** | **CABG, *N (% complete)*** | **AVR, *N (% complete)*** | **TAVI, *N (% complete)*** | **CABG+AVR, *N (% complete)*** |
| --- | --- | --- | --- | --- | --- |
| **Total** | **177,556 (100)** | **39,069 (100)** | **8,028 (100)** | **11,819 (100)** | **4,888 (100)** |
| **Patient characteristic** |  |  |  |  |  |
| Sex | 177,212 (99.81) | 39,069 (100) | 8,028 (100) | 11,819 (100) | 4,888 (100) |
| Age | 177,556 (100) | 39,069 (100) | 8,028 (100) | 11,819 (100) | 4,888 (100) |
| Diabetes mellitus | 173,583 (97.76) | 38,94 (99.67) | 7,985 (99.46) | 11,764 (99.53) | 4,882 (99.88) |
| eGFR | 177,556 (100) | 39,069 (100) | 8,018 (99.88) | 11,779 (99.66) | 4,882 (99.88) |
| Left ventricular ejection fraction | 73,055 (41.14) | 38,857 (99.46) | 8,005 (99.71) | 11,575 (97.94) | 4,864 (99.51) |
| BMI |  | 38,275 (97.97) | 7,982 (99.43) | 11,761 (99.51) | 4,803 (98.26) |
| COPD |  | 39,058 (99.97) | 8,018 (99.88) | 11,79 (99.75) | 4,886 (99.96) |
| Prior cardiac procedure |  | 39,069 (100) | 8,028 (100) | 11,645 (98.53) | 4,888 (100) |
| Multivessel disease | 175,557 (98.87) | 33,887 (86.74) |  |  | 4,144 (84.78) |
| Prior myocardial infarction | 173,495 (97.71) | 39,02 (99.87) |  |  | 4,881 (99.86) |
| Urgency of procedure |  | 39,057 (99.97) | 8,023 (99.94) | 11,75 (99.42) | 4,885 (99.94) |
| Logistic Euroscore I |  | 38,901 (99.57) | 8 (99.65) | 11,231 (95.02) | 4,868 (99.59) |
| Logistic Euroscore II |  | 33,596 (85.99) | 6,93 (86.32) | 9,963 (84.3) | 4,21 (86.13) |
| Active endocarditis |  |  | 8,011 (99.79) |  | 4,884 (99.92) |
| Prior cardio vascular arrest |  | 35,604 (91.13) | 7,497 (93.39) | 11,793 (99.78) | 4,518 (92.43) |
| Chronic total occlusion | 176,319 (99.3) |  |  |  |  |
| Cardiogenic shock | 176,604 (99.46) |  |  |  |  |
| Out of hospital cardiac arrest | 176,63 (99.48) |  |  |  |  |
| Prior CABG | 174,985 (98.55) |  |  |  |  |
| PCI indication (STEMI/NSTEMI/elective) | 177,556 (100) |  |  |  |  |
| NYHA-class IV |  |  |  | 10,898 (92.21) |  |
| **Outcome** | **PCI** | **CABG** | **AVR** | **TAVI** | **CABG+AVR** |
| **Mortality** |  |  |  |  |  |
| Early procedural mortality < 3 days post procedure |  |  |  | 11,819 (100) |  |
| 30-day mortality | 175,93 (99.08) |  |  | 11,785 (99.71) |  |
| 120-day mortality |  | 37,782 (96.71) | 7,825 (97.47) | 113,29 (95.85) | 4,759 (97.36) |
| 1-year mortality (2015-2019) | 150,397 (97.92) | 32,843 (99.63) | 6,859 (99.78) | 9,416 (99.69) | 4,19 (99.76) |
| **Complications** |  |  |  |  |  |
| Cerebro vasculair accident (CVA) with residual injury |  | 38,869 (99.49) | 7,995 (99.59) | 11,317 (95.75) | 4,87 (99.63) |
| Re-exploration < 30 days |  | 37,971 (97.19) | 7,751 (96.55) |  | 4,739 (96.95) |
| Deep sternal wound infection within 30 days |  | 37,968 (97.18) | 7,739 (96.4) |  | 4,736 (96.89) |
| Implantation of new permanent pacemaker within 30 days after procedure |  | 35,253 (90.23) | 4,997 (62.24) | 11,559 (97.8) | 3,064 (62.68) |
| Major vascular complication within 30 days after procedure |  |  |  | 10,274 (86.93) |  |
| Myocardial infarction (STEMI/NSTEMI) within 30 days after procedure | 129,44 (72.90) |  |  |  |  |
| Urgent CABG within 1 day after procedure as result of acute complications | 175,913 (99.07) |  |  |  |  |
| Target vessel revascularization (TVR) within 1 year after procedure | 136,007 (88.56) |  |  |  |  |
| **Reinterventions** |  |  |  |  |  |
| Aortic valve reintervention during follow-up |  |  |  | 10,57 (89.43) | 4,328 (88.54) |
| Coronary reintervention during follow-up |  | 35,253 (89.18) | 7,387 (92.02) |  | 4,321 (88.40) |
