# Supplementary material for: A high-volume study on the impact of diabetes mellitus on clinical outcomes after surgical and percutaneous cardiac interventions
Source: Cardiovasc Diabetol. 2024 Jul 18;23:260. doi: 10.1186/s12933-024-02356-2 (PMC11264856; doi:10.1186/s12933-024-02356-2)
Supplement: Supplementary file 3 — Supplementary Material 3 [file 12933_2024_2356_MOESM3_ESM.docx]

***SUPPLEMENTARY Table 2. Frequency of diabetes mellitus within the study population***

|  |  | **Coronary artery disease (CAD)** | | | | **Aortic Valve Disease** | | | | **Combined CAD + AVD** | |
| --- | --- | --- | --- | --- | --- | --- | --- | --- | --- | --- | --- |
| **Diabetes** | | **PCI**  **n=177,556** | | **CABG**  **N=39,069** | | **AVR**  **N=8,028** | | **TAVI**  **N=11,819** | | **CABG+AVR**  **N=4,888** | |
| **DM** | | 37,512 (21.1%) | | 10,437 (26.7%) | | 1,429 (17.8%) | | 3,258 (27.6%) | | 1,361 (27.8%) | |
|  | **Diet** |  | 424 (0.2%) |  | 307 (0.8%) |  | 72 (0.9%) |  | 154 (1.3%) |  | 39 (0.8%) |
|  | **Oral medication** |  | 11,914 (6.7%) |  | 4,936 (12.6%) |  | 741 (9.2%) |  | 1,626 (13.8%) |  | 706 (14.4%) |
|  | **Insulin** |  | 7474 (4.2%) |  | 3,786 (9.7%) |  | 411 (5.1%) |  | 1,065 (9.0%) |  | 429 (8.8%) |
|  | **Other treatment** |  | 624 (0.4%) |  | 450 (1.2%) |  | 65 (0.8%) |  | 44 (0.4%) |  | 75 (1.5%) |
|  | **No treatment** |  | 968 (0.5%) |  | 238 (0.6%) |  | 32 (0.4%) |  | 67 (0.6%) |  | 30 (0.6%) |
|  | **Treatment unknown** |  | 16,108 (9.1%) |  | 720 (1.8%) |  | 108 (1.3%) |  | 302 (2.6%) |  | 82 (1.7%) |
| **No DM** | | 136,071 (76.6%) | | 28,503 (73.0%) | | 6,556 (81.7%) | | 8,506 (72.6%) | | 3,521 (72.0%) | |
| **Missing** | | 3,973 (2.2%) | | 129 (0.3%) | | 43 (0.5%) | | 55 (0.5%) | | 6 (0.1%) | |
