# Supplementary material for: A high-volume study on the impact of diabetes mellitus on clinical outcomes after surgical and percutaneous cardiac interventions
Source: Cardiovasc Diabetol. 2024 Jul 18;23:260. doi: 10.1186/s12933-024-02356-2 (PMC11264856; doi:10.1186/s12933-024-02356-2)
Supplement: Supplementary file 4 — Supplementary Material 4 [file 12933_2024_2356_MOESM4_ESM.docx]

**SUPPLEMENTARY Table 5. Differences in outcome measures between patients living with diabetes (and different medical treatments) and people living without diabetes for each cardiac procedure after propensity matching**

| **Cardiac disease** | **Cardiac intervention** | **Outcome measure** | **Overall** | **No DM** | **Treatment Unknown** | **No treatment** | **Diet** | **Oral medication** | **Insulin** | **Other treatment** | **p -value** |
| --- | --- | --- | --- | --- | --- | --- | --- | --- | --- | --- | --- |
| **Coronary artery disease (CAD)** | **PCI** | **N** | **114,639** | **76,426** | **16,429** | **980** | **430** | **12,144** | **7,595** | **635** |  |
|  |  | 30-day mortality, N (%) | 3,133 (2.8) | 1,814 (2.4) a, c, d, g | 585 (3.6) a, b | 35 (3.6) | 17 (4.0) | 346 (2.9) b, c, e, f | 309 ( 4.1) d, e | 27 (4.3) f, g | <.001 |
|  |  | 1-year mortality (2015-2019), N(%) | 7,371 (6.8) | 4,208 (5.8) a, b, d, f, i | 1,336 (8.4) a, d, g | 68 (7.6) h | 39 (9.7) b | 816 (7.3) c, d, e | 845 (12.0) e, f, g, h | 59 (9.4) i | <.001 |
|  |  | MI <30 days, N(%) | 666 (.8) | 398 (.7) a | 115 (1.0) a | 11 (1.3) | 1 (.4) b | 67 (.8) b | 74 ( 1.4) | 0 (.0) | <.001 |
|  |  | Urgent CABG < 1 day, N%) | 234 (.2) | 160 (.2) | 36 (.2) | 2 (.2) | 2 (.5) | 20 (.2) | 12 ( .2) | 2 (.3) | .696 |
|  |  | TVR < 1 year (2015-2019), N(%) | 6,186 (6.3) | 3,787 (5.7) a, b, c | 1006 (7.2) a | 48 (5.5) | 22 (5.7) | 684 (6.6) b, d | 579 ( 8.8) d | 60 (9.6) c | <.001 |
|  | **CABG** | **N** | **31,404** | **20,936** | **721** | **239** | **307** | **4,948** | **3,802** | **451** |  |
|  |  | 120-day mortality, N(%) | 648 (2.1) | 384 (1.9) a | 17 (2.4) | 9 (4.0) | 5 (1.7) | 97 (2.0) b | 127 (3.4) a, b | 9 (2.1) | <.001 |
|  |  | 1-year mortality (2015-2019), N(%) | 917 (3.4) | 524 (2.9) a | 26 (4.1) | 11 (5.9) | 8 (3.0) | 152 (3.6) b | 176 (5.4) a, b | 20 (5.2) | <.001 |
|  |  | CVA during admission, N(%) | 226 (.7) | 131 (.6) a | 3 (.4) | 2 (.8) | 3 (1.0) | 42 (.9) | 41 (1.1) a | 4 (.9) | .059 |
|  |  | Re-exploration <30 days, N(%) | 1,235 (4.0) | 841 (4.1) | 23 (3.2) | 15 (6.4) | 10 (3.5) | 175 (3.7) | 153 (4.1) | 18 (4.0) | .615 |
|  |  | DSWI < 30 days, N(%) | 330 (1.1) | 159 (.8) a | 10 (1.4) | 2 (.8) | 5 (1.7) | 60 (1.3) b | 87 (2.4) a, b | 7 (1.6) | <.001 |
| **Aortic valve disease (AVD)** | **AVR** | **N** | **5,740** | **4,305** | **109** | **33** | **73** | **742** | **413** | **65** |  |
|  |  | 120-day mortality, N(%) | 134 (2.4) | 91 (2.2) | 3 (2.8) | 1 ( 3.1) | 3 (4.1) | 19 (2.6) | 16 (4.0) | 1 ( 1.6) | .358 |
|  |  | 1-year mortality (2015-2019), N(%) | 209 (4.1) | 146 (3.9) | 7 (7.8) | 1 ( 4.0) | 3 (4.5) | 30 (4.5) | 19 (5.1) | 3 ( 5.0) | .537 |
|  |  | CVA during admission, N(%) | 55 (1.0) | 40 (.9) | 1 (.9) | 0 ( .0) | 1 (1.4) | 8 (1.1) | 5 (1.2) | 0 ( .0) | .958 |
|  |  | Re-exploration <30 days, N(%) | 318 (5.7) | 239 (5.7) | 7 (6.4) | 5 (15.2) | 5 (7.2) | 38 (5.3) | 23 (5.8) | 1 ( 1.5) | .112 |
|  |  | DSWI < 30 days, N(%) | 31 (.6) | 17 (.4) a | 0 (.0) | 1 ( 3.0) | 0 (.0) | 6 (.8) | 6 (1.5) a | 1 ( 1.5) | .022 |
|  |  | PM < 30 days, N(%) | 152 (4.2) | 119 (4.4) | 2 (1.8) | 0 ( .0) | 2 (3.5) | 15 (3.3) | 11 (4.5) | 3 (15.8) | .142 |
|  | **TAVI** | **N** | **6,558** | **3,279** | **305** | **67** | **155** | **1,635** | **1,073** | **44** |  |
|  |  | Proc. Mortality (3-days), N(%) | 57 ( .9) | 31 ( .9) a | 7 ( 2.3) | 2 ( 3.0) | 0 ( .0) | 10 ( .6) | 6 ( .6) | 1 ( 2.3) | .015 |
|  |  | 30-day mortality, N (%) | 208 ( 3.2) | 106 ( 3.2) | 19 ( 6.3) a, b | 3 ( 4.5) | 3 ( 1.9) | 41 ( 2.5) b | 33 ( 3.1) | 3 ( 6.8) | .022 |
|  |  | 120-day mortality, N(%) | 380 ( 6.1) | 188 ( 6.0) | 24 ( 8.4) | 3 ( 4.8) | 10 ( 6.6) | 81 ( 5.2) | 70 ( 6.8) | 4 ( 9.8) | .299 |
|  |  | 1-year mortality (2015-2019), N(%) | 720 (12.9) a | 331 (11.8) a, b | 46 (19.2) | 6 (12.0) | 18 (12.7) | 179 (12.8) | 132 (14.7) b | 8 (2.0) | .017 |
|  |  | CVA during admission, N(%) | 112 ( 1.8) | 51 ( 1.6) | 7 ( 2.8) | 1 ( 1.5) | 3 ( 2.1) | 26 ( 1.6) | 21 ( 2.0) | 3 ( 6.8) | .293 |
|  |  | PM < 30 days, N(%) | 687 (1.7) | 316 ( 9.8) | 35 (11.6) | 7 (1.4) | 15 ( 9.9) | 177 (11.1) | 129 (12.4) | 8 (18.2) | .156 |
|  |  | Maj. vasc. compl. < 30 days, N(%) | 166 ( 2.9) | 93 ( 3.3) | 7 ( 2.8) | 2 ( 3.0) | 1 ( .9) | 37 ( 2.7) | 25 ( 2.7) | 1 ( 2.4) | .781 |
| **Combined CAD + AVD** | **CABG+AVR** | **N** | **4,089** | **2,726** | **82** | **30** | **39** | **707** | **430** | **75** |  |
|  |  | 120-day mortality, N(%) | 185 (4.6) | 103 (3.9) a | 8 ( 9.8) | 1 (3.4) | 2 (5.4) | 34 (4.9) | 35 ( 8.4) a | 2 (2.8) | .011 |
|  |  | 1-year mortality (2015-2019), N(%) | 239 (6.6) | 138 (5.7) a | 8 (1.5) | 1 (4.0) | 3 (8.8) | 45 (7.2) | 41 (11.1) a | 3 (4.9) | .056 |
|  |  | CVA during admission, N(%) | 69 (1.7) | 43 (1.6) | 2 ( 2.4) | 0 (.0) | 1 (2.6) | 19 (2.7) | 3 ( .7) | 1 (1.3) | .301 |
|  |  | Re-exploration <30 days, N(%) | 316 (8.0) | 200 (7.5) | 8 ( 9.8) | 1 (3.3) | 3 (8.6) | 54 (7.9) | 44 (1.6) | 6 (8.0) | .106 |
|  |  | DSWI < 30 days, N(%) | 41 (1.0) | 21 (.8) a | 0 ( .0) | 0 (.0) | 1 (2.9) | 9 (1.3) | 10 ( 2.4) a | 0 (.0) | .034 |
|  |  | PM < 30 days, N(%) | 86 (3.4) | 64 (3.7) | 0 ( .0) | 0 (.0) | 0 (.0) | 14 (3.1) | 8 ( 3.1) | 0 (.0) | .488 |

** Two similar letters per row have a statistically significant difference (p<.05) after Bonferroni correction, proc. Mortality (3-days) = procedural mortality within 3 days, PM < 30-days = implantation of new permanent pacemaker within 30 days, maj. vasc. compl < 30-days = major vascular complication within 30 days, MI < 30 days = myocardial infarction within 30 days, TVR < 1 year = Target Vessel Revascularization within 1 year. An overview of the available baseline characteristics per procedure is shown in Table 1 of the Supplementary materials.*
