# Supplementary material for: A high-volume study on the impact of diabetes mellitus on clinical outcomes after surgical and percutaneous cardiac interventions
Source: Cardiovasc Diabetol. 2024 Jul 18;23:260. doi: 10.1186/s12933-024-02356-2 (PMC11264856; doi:10.1186/s12933-024-02356-2)
Supplement: Supplementary file 5 — Supplementary Material 5 [file 12933_2024_2356_MOESM5_ESM.docx]

**SUPPLEMENTARY Table 4. Differences in outcome measures between the two study groups after propensity matching for each cardiac procedure**

| **Cardiac disease** | **Cardiac intervention** | **Outcome measure** | **Overall** | **DM** | **no DM** | **p-value** | **OR (95% CI)** |
| --- | --- | --- | --- | --- | --- | --- | --- |
| **Coronary artery disease (CAD)** | **PCI** | **N** | **114,639** | **38,213** | **76,426** |  |  |
|  |  | 30-day mortality, N (%) | 3,133 (2.8) | 1,319 (3.5) | 1,814 (2.4) | <.001 | 1.47 (1.37 - 1.58) |
|  |  | 1-year mortality (2015-2019), N(%) | 7,371 (6.8) | 3,163 (8.7) | 4,208 (5.8) | <.001 | 1.54 (1.47 - 1.62) |
|  |  | MI <30 days, N(%) | 666 (0.8) | 268 (1.0) | 398 (0.7) | <.001 | 1.39 (1.19 - 1.62) |
|  |  | Urgent CABG < 1 day, N%) | 234 (0.2) | 74 (0.2) | 160 (0.2) | .632 | .93 (.70 - 1.21) |
|  |  | TVR < 1 year (2015-2019), N(%) | 6,186 (6.3) | 2,399 (7.3) | 3,787 (5.7) | <.001 | 1.30 (1.23 - 1.37) |
|  | **CABG** | **N** | **31,404** | **10,468** | **20,936** |  |  |
|  |  | 120-day mortality, N(%) | 648 (2.1) | 264 (2.6) | 384 (1.9) | <.001 | 1.39 [1.18, 1.63] |
|  |  | 1-year mortality (2015-2019), N(%) | 917 (3.4) | 393 (4.4) | 524 (2.9) | <.001 | 1.55 [1.35, 1.77] |
|  |  | CVA during admission, N(%) | 226 (0.7) | 95 (0.9) | 131 (0.6) | .006 | 1.46 [1.12, 1.90] |
|  |  | Re-exploration <30 days, N(%) | 1,235 (4.0) | 394 (3.9) | 841 (4.1) | .348 | .94 [.83, 1.06] |
|  |  | DSWI < 30 days, N(%) | 330 (1.1) | 171 (1.7) | 159 (0.8) | <.001 | 2.19 [1.76, 2.72] |
| **Aortic valve disease (AVD)** | **AVR** | **N** | **5,740** | **1,435** | **4,305** |  |  |
|  |  | 120-day mortality, N(%) | 134 (2.4) | 43 (3.1) | 91 (2.2) | .075 | 1.42 (.98 - 2.04) |
|  |  | 1-year mortality (2015-2019), N(%) | 209 (4.1) | 63 (4.9) | 146 (3.9) | .116 | 1.29 (.95 - 1.74) |
|  |  | CVA during admission, N(%) | 55 (1.0) | 15 (1.0) | 40 (0.9) | .817 | 1.13 (.60 - 2.00) |
|  |  | Re-exploration <30 days, N(%) | 318 (5.7) | 79 (5.7) | 239 (5.7) | .997 | .99 (.76 - 1.28) |
|  |  | DSWI < 30 days, N(%) | 31 (0.6) | 14 (1.0) | 17 (0.4) | .017 | 2.48 (1.20 - 5.05) |
|  |  | PM < 30 days, N(%) | 152 (4.2) | 33 (3.7) | 119 (4.4) | .422 | .84 (.56 - 1.22) |
|  | **TAVI** | **N** | **6,558** | **3,279** | **3,279** |  |  |
|  |  | proc. Mortality (3-days), N(%) | 57 ( 0.9) | 26 ( 0.8) | 31 ( 0.9) | .595 | .84 (.49 - 1.41) |
|  |  | 30-day mortality, N (%) | 208 ( 3.2) | 102 ( 3.1) | 106 ( 3.2) | .836 | .96 (.73 - 1.27) |
|  |  | 120-day mortality, N(%) | 380 ( 6.1) | 192 ( 6.1) | 188 ( 6.0) | .866 | 1.02 (.83 - 1.26) |
|  |  | 1-year mortality (2015-2019), N(%) | 720 (12.9) | 389 (14.0) | 331 (11.8) | .017 | 1.21 (1.04 - 1.42) |
|  |  | CVA during admission, N(%) | 112 ( 1.8) | 61 ( 2.0) | 51 ( 1.6) | .363 | 1.21 (.83 - 1.77) |
|  |  | PM < 30 days, N(%) | 687 (10.7) | 371 (11.6) | 316 ( 9.8) | .023 | 1.21 (1.03 - 1.41) |
|  |  | Maj. vasc. compl. < 30 days, N(%) | 166 ( 2.9) | 73 ( 2.6) | 93 ( 3.3) | .178 | .89 (.65 - 1.12) |
| **Combined CAD + AVD** | **CABG+AVR** | **N** | **4,089** | **1,363** | **2,726** |  |  |
|  |  | 120-day mortality, N(%) | 185 (4.6) | 82 (6.2) | 103 (3.9) | .002 | 1.63 (1.21 - 2.19) |
|  |  | 1-year mortality (2015-2019), N(%) | 239 (6.6) | 101 (8.4) | 138 (5.7) | .002 | 1.52 (1.16 - 1.98) |
|  |  | CVA during admission, N(%) | 69 (1.7) | 26 (1.9) | 43 (1.6) | .519 | 1.21 (.73 - 1.97) |
|  |  | Re-exploration <30 days, N(%) | 316 (8.0) | 116 (8.8) | 200 (7.5) | .194 | 1.18 (.93 - 1.50) |
|  |  | DSWI < 30 days, N(%) | 41 (1.0) | 20 (1.5) | 21 (0.8) | .051 | 1.92 (1.03 - 3.57) |
|  |  | PM < 30 days, N(%) | 86 (3.4) | 22 (2.6) | 64 (3.7) | .181 | .70 (.42 - 1.12) |

**proc. Mortality (3-days) = procedural mortality within 3 days, PM < 30-days = implantation of new permanent pacemaker within 30 days, maj. vasc. compl < 30-days = major vascular complication within 30 days, MI < 30 days = myocardial infarction within 30 days, TVR < 1 year = Target Vessel Revascularization within 1 year. An overview of the available baseline characteristics per procedure is shown in Table 1 of the Supplementary materials.*
