# Supplementary material for: A high-volume study on the impact of diabetes mellitus on clinical outcomes after surgical and percutaneous cardiac interventions
Source: Cardiovasc Diabetol. 2024 Jul 18;23:260. doi: 10.1186/s12933-024-02356-2 (PMC11264856; doi:10.1186/s12933-024-02356-2)
Supplement: Supplementary file 6 — Supplementary Material 6 [file 12933_2024_2356_MOESM6_ESM.docx]

**SUPPLEMENTARY Table 3. Differences in patient characteristics between the two study groups after propensity matching**

| **Cardiac disease** | **Cardiac intervention** | **Outcome measure** | **Overall** | **DM** | **no DM** | **p-value** | **SMD** |
| --- | --- | --- | --- | --- | --- | --- | --- |
| **Coronary artery disease (CAD)** | **PCI (1:2)** | **N** | **114,639** | **38,213** | **76,426** |  |  |
|  |  | Sex (female), n (%) | 36,136 (31.5) | 12,043 (31.5) | 24,093 (31.5) | .980 | <.001 |
|  |  | Age, median (IQR) | 70.00 [61.00, 77.00] | 70.00 [62.00, 76.00] | 70.00 [61.00, 77.00] | .001 | .019 |
|  |  | eGFR, median (IQR) | 86.00 [72.00, 106.00] | 87.00 [71.00, 109.00] | 85.00 [72.00, 104.00] | <.001 | .053 |
|  |  | Multi vessel dis., n (%) | 66,192 (57.7) | 22,109 (57.9) | 44,083 (57.7) | .572 | .004 |
|  |  | Prior MI, n (%) | 33,260 (29.0) | 11,226 (29.4) | 22,034 (28.8) | .055 | .012 |
|  |  | CTO, n (%) | 7,379 ( 6.4) | 2,453 ( 6.4) | 4,926 ( 6.4) | .875 | .001 |
|  |  | shock, n (%) | 2,506 ( 2.2) | 895 ( 2.3) | 1,611 ( 2.1) | .011 | .016 |
|  |  | OHCA, n (%) | 2,491 ( 2.2) | 897 ( 2.3) | 1,594 ( 2.1) | .004 | .018 |
|  |  | Prior CABG, n (%) | 16,134 (14.1) | 5,853 (15.3) | 10,281 (13.5) | <.001 | .053 |
|  |  | PCI (STEMI), n (%) | 22,351 (19.5) | 7,746 (20.3) | 14,605 (19.1) | <.001 | .029 |
|  | **CABG (1:2)** | **N** | **31,404** | **10,468** | **20,936** |  |  |
|  |  | Sex (female), n (%) | 6,647 ( 21.2) | 2,394 ( 22.9) | 4,253 ( 20.3) | <.001 | .062 |
|  |  | Age, median (IQR) | 68.00 [61.00, 74.00] | 68.00 [61.00, 74.00] | 68.00 [61.00, 74.00] | .809 | .006 |
|  |  | eGFR, median (IQR) | 87.00 [75.00, 101.00] | 87.00 [73.00, 105.00] | 86.00 [75.00, 100.00] | .116 | .097 |
|  |  | LVEF, median (IQR) | 55.00 [43.00, 55.00] | 55.00 [40.00, 55.00] | 55.00 [43.00, 55.00] | .001 | .050 |
|  |  | BMI, median (IQR) | 27.97 [25.51, 30.80] | 28.40 [25.67, 31.64] | 27.76 [25.46, 30.41] | .006 | .078 |
|  |  | COPD, n (%) | 3,342 ( 10.6) | 1,218 ( 11.6) | 2,124 ( 10.1) | <.001 | .048 |
|  |  | Prior card. Pro., n (%) | 533 ( 1.7) | 186 ( 1.8) | 347 ( 1.7) | .468 | .009 |
|  |  | Multi vessel dis., n (%) | 28,549 ( 90.9) | 9,514 ( 90.9) | 19,035 ( 90.9) | .939 | .001 |
|  |  | Prior MI, n (%) | 10,812 ( 34.4) | 3,590 ( 34.3) | 7,222 ( 34.5) | .734 | .004 |
|  |  | Urgent procedure, n (%) | 13,549 ( 43.1) | 4,521 ( 43.2) | 9,028 ( 43.1) | .920 | .001 |
|  |  | Log. EuroSCORE I, median (IQR) | 3.04 [1.73, 5.58] | 3.16 [1.85, 5.89] | 2.97 [1.68, 5.43] | <.001 | .045 |
|  |  | Prior CVA, n (%) | 1,893 ( 6.0) | 731 ( 7.0) | 1,162 ( 5.6) | <.001 | .059 |
| **Aortic valve disease (AVD)** | **AVR (1:3)** | **N** | **5,740** | **1,435** | **4,305** |  |  |
|  |  | Sex (female), n (%) | 2,141 (37.3) | 547 (38.1) | 1,594 (37.0) | .478 | .023 |
|  |  | Age, median (IQR) | 71.00 [65.00, 76.00] | 71.00 [65.00, 75.00] | 71.00 [65.00, 76.00] | .236 | .010 |
|  |  | eGFR, median (IQR) | 84.00 [72.00, 99.00] | 83.00 [71.00, 100.00] | 84.00 [72.00, 98.00] | .681 | .027 |
|  |  | LVEF, median (IQR) | 55.00 [55.00, 59.00] | 55.00 [55.00, 58.50] | 55.00 [55.00, 59.00] | .912 | .007 |
|  |  | BMI, median (IQR) | 29.07 [26.37, 32.43] | 29.37 [26.34, 32.52] | 29.06 [26.27, 32.36] | .212 | .024 |
|  |  | COPD, n (%) | 823 (14.3) | 218 (15.2) | 605 (14.1) | .307 | .032 |
|  |  | Prior card. pro., n (%) | 505 ( 8.8) | 131 ( 9.1) | 374 ( 8.7) | .647 | .015 |
|  |  | Urgent procedure, n (%) | 743 (12.9) | 192 (13.4) | 551 (12.8) | .602 | .017 |
|  |  | Log. EuroSCORE I, median (IQR) | 5.09 [3.30, 7.87] | 5.15 [3.40, 8.05] | 5.06 [3.27, 7.81] | .226 | .030 |
|  |  | Active endocarditis, n (%) | 296 ( 5.2) | 77 ( 5.4) | 219 ( 5.1) | .730 | .013 |
|  |  | Prior CVA, n (%) | 384 ( 6.7) | 98 ( 6.8) | 286 ( 6.6) | .855 | .007 |
|  | **TAVI (1:1)** | **N** | **6,558** | **3,279** | **3,279** |  |  |
|  |  | Sex (female), n (%) | 3,068 (46.8) | 1,546 (47.1) | 1,522 (46.4) | .569 | .015 |
|  |  | Age, median (IQR) | 79.00 [74.00, 83.00] | 79.00 [74.00, 83.00] | 80.00 [74.00, 83.00] | .046 | .013 |
|  |  | eGFR, median (IQR) | 95.00 [77.00, 121.00] | 97.00 [78.00, 125.00] | 93.00 [77.00, 117.00] | <.001 | .041 |
|  |  | LVEF, median (IQR) | 55.00 [40.00, 55.00] | 55.00 [40.00, 55.00] | 55.00 [40.00, 55.00] | .946 | .006 |
|  |  | BMI, median (IQR) | 28.30 [25.15, 32.05] | 28.35 [25.25, 32.18] | 28.18 [25.02, 32.03] | .139 | .046 |
|  |  | COPD, n (%) | 1,420 (21.7) | 720 (22.0) | 700 (21.3) | .569 | .015 |
|  |  | Prior card. pro., n (%) | 1,450 (22.1) | 711 (21.7) | 739 (22.5) | .422 | .021 |
|  |  | Log. EuroSCORE I, median (IQR) | 11.81 [7.94, 19.00] | 12.07 [8.00, 19.26] | 11.54 [7.85, 18.62] | .085 | .038 |
|  |  | Prior CVA, n (%) | 769 (11.7) | 381 (11.6) | 388 (11.8) | .818 | .007 |
|  |  | NYHA-class IV, n (%) | 490 ( 7.5) | 248 ( 7.6) | 242 ( 7.4) | .814 | .007 |
| **Combined CAD + AVD** | **CABG+AVR** | **N** | **4,089** | **1,363** | **2,726** |  |  |
|  |  | Sex (female), n (%) | 939 (23.0) | 327 (24.0) | 612 (22.5) | .287 | .036 |
|  |  | age, median (IQR) | 72.00 [67.00 - 77.00] | 72.00 [68.00 - 76.00] | 72.00 [67.00 - 77.00] | .670 | .013 |
|  |  | eGFR, median (IQR) | 88.00 [75.00 - 105.00] | 90.00 [74.00 - 110.00] | 87.00 [76.00 - 102.00] | .002 | .097 |
|  |  | LVEF, median (IQR) | 55.00 [44.00 - 56.00] | 55.00 [43.00 - 56.00] | 55.00 [45.00 - 56.00] | .988 | .017 |
|  |  | BMI, median (IQR) | 28.72 [25.96 - 31.74] | 28.72 [26.03 - 31.77] | 28.73 [25.88 - 31.74] | .841 | .023 |
|  |  | COPD, n (%) | 626 (15.3) | 215 (15.8) | 411 (15.1) | .591 | .019 |
|  |  | Prior card. pro., n (%) | 118 ( 2.9) | 42 ( 3.1) | 76 ( 2.8) | .668 | .017 |
|  |  | Multi vessel dis., n (%) | 2633 (64.4) | 875 (64.2) | 1758 (64.5) | .881 | .006 |
|  |  | Prior MI, n (%) | 519 (12.7) | 181 (13.3) | 338 (12.4) | .455 | .026 |
|  |  | Urgent procedure, n (%) | 1,021 (25.0) | 344 (25.2) | 677 (24.8) | .808 | .009 |
|  |  | Log. EuroSCORE I, median (IQR) | 5.85 [3.73 - 9.57] | 6.04 [3.86 - 10.06] | 5.71 [3.70 - 9.28] | .006 | .089 |
|  |  | Active endocarditis, n (%) | 55 ( 1.3) | 19 ( 1.4) | 36 ( 1.3) | .962 | .006 |
|  |  | Prior CVA, n (%) | 319 ( 7.8) | 111 ( 8.1) | 208 ( 7.6) | .606 | .019 |

**eGFR =Estimated Glomerular Filtration Rate, LVEF = Left ventricular ejection fraction, BMI = body mass index, COPD = chronic obstructive pulmonary disease, Prior. Card. Pro. = prior cardiac procedure, Multi vessel dis. = multi vessel disease, prior MI = prior myocardial infarction, log. EuroSCORE I = logistic EuroSCORE I, log. EuroSCORE II = logistic EuroSCORE II, prior CVA = cerebrovascular accident, OHCA = out of hospital arrest. An overview of the available baseline characteristics per procedure is shown in Table 1 of the Supplementary materials.*
