# Supplementary material for: A high-volume study on the impact of diabetes mellitus on clinical outcomes after surgical and percutaneous cardiac interventions
Source: Cardiovasc Diabetol. 2024 Jul 18;23:260. doi: 10.1186/s12933-024-02356-2 (PMC11264856; doi:10.1186/s12933-024-02356-2)
Supplement: Supplementary file 7 — Supplementary Material 7 [file 12933_2024_2356_MOESM7_ESM.docx]

***SUPPLEMENTARY TABLE 6b. Results of the multivariate logistic regression analyses with correlation between diabetes mellitus and all early outcomes (cohort 2018-2020)***

|  | ***Coronary artery disease (CAD)*** | | | | ***Aortic valve disease (AVD)*** | | | | ***Combined CAD and AVD*** | |
| --- | --- | --- | --- | --- | --- | --- | --- | --- | --- | --- |
|  | ***PCI*** | | ***CABG*** | | ***AVR*** | | ***TAVI*** | | ***CABG+AVR*** | |
| ***Outcome measure*** | ***OR (95% CI)*** | ***p-value*** | ***OR (95% CI)*** | ***p-value*** | ***OR (95% CI)*** | ***p-value*** | ***OR (95% CI)*** | ***p-value*** | ***OR (95% CI)*** | ***p-value*** |
| ***Mortality*** |  |  |  |  |  |  |  |  |  |  |
| ***Proc. mortality (3-days)*** | n/a | n/a | n/a | n/a | n/a | n/a | .74 (.39-1.38) | .34 | n/a | n/a |
| ***30-day mortality*** | 1.71 (1.54-1.90) | <.001 | n/a | n/a | n/a | n/a | .94 (.67-1.33) | .74 | n/a | n/a |
| ***120-day mortality*** | n/a | n/a | 1.27 (1.00-1.60) | .05 | 1.10 (.61-1.98) | .75 | 1.27 (.99-1.62) | .06 | 1.95 (1.28-2.98) | .002 |
| ***1-year mortality (2015-2019)*** | 1.70 (1.57-1.84) | <.001 | 1.36 (1.08-1.72) | .01 | .84 (.45-1.57) | .59 | 1.36 (1.10-1.68) | .005 | 1.58 (.98-2.55) | .06 |
|  |  |  |  |  |  |  |  |  |  |  |
| ***Complications*** |  |  |  |  |  |  |  |  |  |  |
| ***CVA during admission*** | n/a | n/a | 1.12 (.77-1.64) | .55 | 1.43 (.67-3.04) | .36 | .97 (.67-1.42) | .89 | .88 (.44-1.75) | .71 |
| ***Re-exploration <30 days*** | n/a | n/a | .95 (.81-1.11) | .49 | .80 (.53-1.19) | .27 | n/a | n/a | 1.16 (.83-1.61) | .39 |
| ***DSWI < 30 days*** | n/a | n/a | 2.28 (1.71-3.04) | <.001 | 1.47 (.47-4.60) | .51 | n/a | n/a | 2.06 (.85-4.99) | .11 |
| ***PM < 30 days*** | n/a | n/a | n/a | n/a | .85 (.45-1.59) | .60 | 1.18 (.99-1.41) | .06 | .78 (.31-1.92) | .59 |
| ***Maj. vasc. compl. < 30 days*** | n/a | n/a | n/a | n/a | n/a | n/a | .91 (.64-1.30) | .61 | n/a | n/a |
| ***MI <30 days*** | 1.47 (1.21-1.78) | <.001 | n/a | n/a | n/a | n/a | n/a | n/a | n/a | n/a |
| ***Urgent CABG < 1 day*** | .63 (.41-.97) | .04 | n/a | n/a | n/a | n/a | n/a | n/a | n/a | n/a |
| ***TVR < 1 year (2015-2019)*** | 1.29 (1.19-1.39) | <.001 | n/a | n/a | n/a | n/a | n/a | n/a | n/a | n/a |

** n/a = not applicable (not part of NHR indicators), proc. Mortality (3-days) = procedural mortality within 3 days, PM < 30-days = implantation of new permanent pacemaker within 30 days, maj. vasc. compl < 30-days = major vascular complication within 30 days, MI < 30 days = myocardial infarction within 30 days, TVR < 1 year = Target Vessel Revascularization within 1 year. An overview of the available baseline characteristics per procedure is shown in Table 1 of the Supplementary materials.*
