# Supplementary material for: A high-volume study on the impact of diabetes mellitus on clinical outcomes after surgical and percutaneous cardiac interventions
Source: Cardiovasc Diabetol. 2024 Jul 18;23:260. doi: 10.1186/s12933-024-02356-2 (PMC11264856; doi:10.1186/s12933-024-02356-2)
Supplement: Supplementary file 8 — Supplementary Material 8 [file 12933_2024_2356_MOESM8_ESM.docx]

**SUPPLEMENTARY Table 6a. *Results of the multivariate logistic regression analyses with correlation between diabetes mellitus and all early outcomes (cohort 2015-2017)***

|  | ***Coronary artery disease (CAD)*** | | | | ***Aortic valve disease (AVD)*** | | | | ***Combined CAD and AVD*** | |
| --- | --- | --- | --- | --- | --- | --- | --- | --- | --- | --- |
|  | ***PCI*** | | ***CABG*** | | ***AVR*** | | ***TAVI*** | | ***CABG+AVR*** | |
| ***Outcome measure*** | ***OR (95% CI)*** | ***p-value*** | ***OR (95% CI)*** | ***p-value*** | ***OR (95% CI)*** | ***p-value*** | ***OR (95% CI)*** | ***p-value*** | ***OR (95% CI)*** | ***p-value*** |
| ***Mortality*** |  |  |  |  |  |  |  |  |  |  |
| ***Proc. mortality (3-days)*** | n/a | n/a | n/a | n/a | n/a | n/a | .71 (.37-1.35) | .29 | n/a | n/a |
| ***30-day mortality*** | 1.62 (1.45-1.82) | <.001 | n/a | n/a | n/a | n/a | 1.13 (.80-1.58) | .49 | n/a | n/a |
| ***120-day mortality*** | n/a | n/a | 1.43 (1.15-1.77) | <.001 | 1.85 (1.15-2.98) | .01 | 1.00 (.76-1.31) | .97 | 1.07 (.71-1.63) | .74 |
| ***1-year mortality (2015-2019)*** | 1.66 (1.54-1.78) | <.001 | 1.47 (1.23-1.77) | <.001 | 1.42 (.97-2.09) | .07 | 1.11 (.91-1.35) | .29 | 1.07 (.75-1.54) | .71 |
|  |  |  |  |  |  |  |  |  |  |  |
| ***Complications*** |  |  |  |  |  |  |  |  |  |  |
| ***CVA during admission*** | n/a | n/a | 1.51 (1.07-2.14) | .020 | .87 (.35-2.13) | .76 | 1.22 (.73-2.02) | .45 | 1.18 (.63-2.20) | .60 |
| ***Re-exploration <30 days*** | n/a | n/a | .89 (.75-1.06) | .187 | 1.18 (.85-1.64) | .32 | n/a | n/a | 1.13 (.82-1.57) | .46 |
| ***DSWI < 30 days*** | n/a | n/a | 2.06 (1.52-2.80) | <.001 | 4.99 (1.94-12.82) | <.001 | n/a | n/a | 1.87 (.84-4.18) | .13 |
| ***PM < 30 days*** | n/a | n/a | n/a | n/a | .89 (.55-1.46) | .65 | 1.21 (.99-1.48) | .07 | .61 (.35-1.09) | .10 |
| ***Maj. vasc. compl. < 30 days*** | n/a | n/a | n/a | n/a | n/a | n/a | .71 (.46-1.09) | .11 | n/a | n/a |
| ***MI <30 days*** | 1.40 (1.11-1.75) | .005 | n/a | n/a | n/a | n/a | n/a | n/a | n/a | n/a |
| ***Urgent CABG < 1 day*** | 1.24 (.89-1.73) | .21 | n/a | n/a | n/a | n/a | n/a | n/a | n/a | n/a |
| ***TVR < 1 year (2015-2019)*** | 1.32 (1.22-1.42) | <.001 | n/a | n/a | n/a | n/a | n/a | n/a | n/a | n/a |

** n/a = not applicable (not part of NHR indicators), proc. Mortality (3-days) = procedural mortality within 3 days, PM < 30-days = implantation of new permanent pacemaker within 30 days, maj. vasc. compl < 30-days = major vascular complication within 30 days, MI < 30 days = myocardial infarction within 30 days, TVR < 1 year = Target Vessel Revascularization within 1 year. An overview of the available baseline characteristics per procedure is shown in Table 1 of the Supplementary materials.*
