# Supplementary material for: A high-volume study on the impact of diabetes mellitus on clinical outcomes after surgical and percutaneous cardiac interventions
Source: Cardiovasc Diabetol. 2024 Jul 18;23:260. doi: 10.1186/s12933-024-02356-2 (PMC11264856; doi:10.1186/s12933-024-02356-2)
Supplement: Supplementary file 9 — Supplementary Material 9 [file 12933_2024_2356_MOESM9_ESM.pdf]

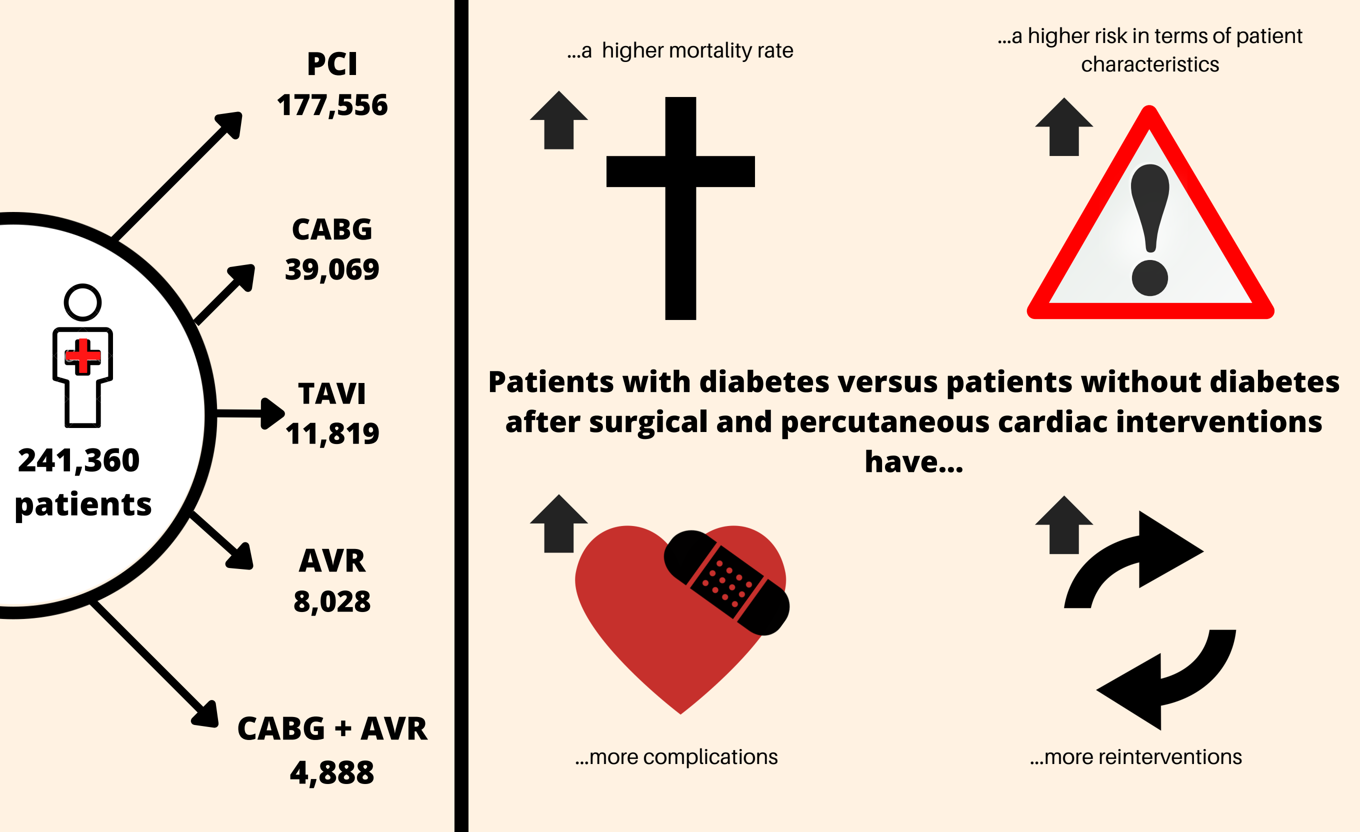
PCI
177,556
CABG
39,069
TAVI
11,819
AVR
8,028
CABG + AVR
4,888
241,360
patients
Patients with diabetes versus patients without diabetes after surgical and percutaneous cardiac interventions have...
...a higher mortality rate
...a higher risk in terms of patient characteristics
...more complications
...more reinterventions
